# Linkage to care and prevention after HIV self‐testing: a systematic review and meta‐analysis

**DOI:** 10.1002/jia2.26388

**Published:** 2024-12-11

**Authors:** Ying Zhang, Su Mei Goh, James Tapa, Cheryl C. Johnson, Eric P. F. Chow, Lei Zhang, Tiffany Phillips, Christopher K. Fairley, Jason J. Ong

**Affiliations:** ^1^ School of Translational Medicine Monash University Melbourne Victoria Australia; ^2^ Melbourne Sexual Health Centre The Alfred Hospital Melbourne Victoria Australia; ^3^ Chelsea and Westminster Trust London UK; ^4^ Global HIV, Hepatitis and STI Programmes World Health Organization Geneva Switzerland; ^5^ Centre for Epidemiology and Biostatistics Melbourne School of Population and Global Health, The University of Melbourne Melbourne Victoria Australia; ^6^ Clinical Research Department London School of Hygiene and Tropical Medicine London UK

**Keywords:** ART, confirmatory testing, HIV, linkage, PrEP, self‐test

## Abstract

**Introduction:**

Effective linkage to prevention and care is a crucial step following HIV testing services. This systematic review aimed to determine the proportion of individuals linked to prevention and care after HIV self‐testing (HIVST) and describe factors associated with linkage.

**Methods:**

Following PRISMA guidelines, a comprehensive search across eight databases (2010–October 2023) identified studies on linkage to care after HIVST, defined as receiving a confirmatory test or initiating antiretroviral therapy (ART) if the self‐test was reactive, and/or pre‐exposure prophylaxis (PrEP) if the self‐test was non‐reactive. A random‐effects meta‐analysis summarized the findings and meta‐regression explored study‐level covariates, such as world region, population type and service delivery model, that might explain the between‐study heterogeneity.

**Results:**

From 10,071 screened studies, 173 were included in the meta‐analysis. The majority of studies focused on key populations in Africa using unassisted, oral fluid‐based HIVST kits. Among those with reactive HIVST results, 92% (95% confidence interval [CI]: 88–95) were linked to confirmatory testing (*n* = 124 studies), and 89% (95% CI: 84–93) of newly diagnosed individuals initiated ART (*n* = 88 studies). Overall, 84% (95% CI: 74–93) of self‐testers were linked to care (*n* = 69 studies). However, only 9% (95% CI: 2–19) of individuals with non‐reactive HIVST results were linked to PrEP services (*n* = 9 studies). Assisted HIVST was associated with higher linkage rates to confirmatory testing and ART initiation compared to unassisted testing. Meta‐regression revealed that the type of delivery model for the HIVST kits influenced linkage and that individuals who obtained their HIVST kits through a social network‐based approach (SNA) were more likely to be linked to confirmatory testing (adjusted odds ratio = 1.28 [95% CI: 1.10–1.50], *p* = 0.001) compared to non‐SNA service delivery model.

**Discussion:**

In the context of expanding HIVST services globally, we found that linkage to confirmatory testing and ART initiation after HIVST is generally high, particularly when assisted HIVST or SNA‐based distribution is used.

**Conclusions:**

Strengthening timely linkage is vital for improving health outcomes, reducing HIV transmission and achieving the UNAIDS 95‐95‐95 goal. Ongoing research and collaboration with community‐based organizations are needed to overcoming barriers and ensuring positive outcomes for those using HIVST.

**PROSPERO Number:**

CRD42022357570.

## 1 INTRODUCTION

HIV testing is central to the global strategy to control the HIV pandemic and achieve the Joint United Nations Programme on HIV and AIDS (UNAIDS) 95‐95‐95 target by 2030 [1]. In 2021, 85% of people living with HIV (PLHIV) globally were aware of their HIV status and 75% received antiretroviral therapy (ART), but about 5.9 million people remained undiagnosed, with those in this group less likely to use traditional facility‐based HIV testing services [1]. In 2016, the World Health Organisation (WHO) strongly recommended HIV self‐testing (HIVST) as an additional testing approach to reverse the inequities in access to HIV testing facing key populations [2]. Since then, to increase early detection and awareness of HIV acquisition, HIVST has emerged as a promising approach, particularly for its ability to reach individuals underserved by the HIV testing strategies currently available [3], thereby contributing to the global efforts towards HIV prevention and treatment. Prior research has demonstrated that HIVST can increase community levels of HIV testing [4], promote partner testing [5] and higher ART initiations [6]. However, the success of HIVST programmes hinges not only on accurate and accessible testing methods, but also on effective linkages to subsequent care and treatment services.

Despite the growing recognition of HIVST as a valuable tool in the fight against HIV, there remains a critical gap in understanding the outcomes and challenges associated with linking individuals to appropriate care post‐HIVST. The WHO defines linkage to care as the time from HIV diagnosis to enrolment in HIV care or treatment [2]. Timely linkage to care is critical for successful HIV treatment and care and has a direct impact on survival and the onward transmission of HIV. PLHIV should be promptly linked to care following HIVST to facilitate the initiation of ART and prevent onward transmission to subsequent partners. Similarly, it is important to optimize the linkage between individuals who had a non‐reactive test but continue to be at risk and highly effective HIV prevention methods, such as pre‐exposure prophylaxis (PrEP). As such, it is critical to monitor and measure linkages to care so that missed opportunities to link newly diagnosed people and at‐risk individuals to HIV care are identified and gaps can be closed. However, measuring or monitoring linkages to HIV care following reactive HIVST results can be challenging given the private nature of HIVST [7].

While current research primarily focuses on developing a better understanding of HIVST effectiveness and devising innovative techniques for targeting hard‐to‐reach persons, more research is needed to understand the challenges and facilitators of linking individuals to HIV care following a reactive HIVST, which is crucial to improving HIVST programmes. A systematic review of randomized controlled trials (RCTs) reporting linkage to care following HIVST found comparable linkage rates between the HIVST arm and the standard‐of‐care arm [8]. However, RCTs are conducted under controlled settings, and hence do not reflect real‐world situations. Collating and synthesizing all available data (i.e. different study designs) may facilitate an understanding of the structural and individual variables that obstruct the linkage to HIV care after a reactive HIVST result, and it may also guide programme implementation, identify research needs and influence policy development.

This review aims to synthesize and critically appraise the existing evidence regarding post‐HIVST linkages to care. We sought to collate the proportion of individuals linked to care (i.e. confirmatory testing, ART initiation) or prevention (i.e. PrEP) following the use of an HIVST kit and explore the underlying facilitators of and barriers to linking HIVST users to care or prevention. The findings of this review are expected to inform policymakers, healthcare providers and programme implementers on strategies to enhance the effectiveness of HIVST initiatives by ensuring a seamless and efficient linkage to follow‐up care.

## 2 METHODS

This review follows the recommendations in the *Cochrane Handbook for Systematic Reviews* [9] and is reported according to the preferred reporting items for systematic reviews and meta‐analyses (PRISMA) guidelines [10]. Eight databases (Medline, Embase, Global Health, PsycINFO, Cochrane CENTRAL, Social Work Abstracts, CINAHL and Web of Science) were searched on 11 September 2022 and updated on 01 October 2023. The strategy was built around overarching terms such as “HIV” and “self‐testing” and adapted to each database (Table S1). Studies published from January 2010 to October 2023 were included, and no language restrictions were set. Reference lists were assessed to identify any other relevant papers, and conference abstract searches included the Conference on Retroviruses and Opportunistic Infections, the International AIDS Conference and the International AIDS Society Conference. Studies were included if they contained primary data on linkages to care or prevention post‐HIVST. Articles without primary data, reviews and protocol papers were excluded, after which three reviewers (YZ, SMG, JT) independently screened the remaining titles and abstracts and another reviewer (JJO) resolved discrepancies. Non‐English‐language studies were translated and excluded if they did not meet the inclusion criteria, and after the full‐text screening, YZ, SMG and JT independently extracted data from the included studies using a data extraction spreadsheet and JJO checked the data. We extracted data on the study type (RCT, non‐RCT studies), service delivery models for HIVST access (pharmacy‐, community‐ or facility‐based; peer‐educator; online; antenatal care), type of HIVST (blood‐based vs oral‐fluid), study population (key population such as men who have sex with men [MSM], female sex workers vs. non‐key population) and assistance during HIVST (i.e. assisted self‐testing). We also extracted data on the time taken between testing and the linkage to any health services for individuals reporting reactive HIVST results. Studies that reported from the same trial but conducted over different time periods were considered as separate studies. For studies with multiple arms that focused exclusively on HIVST, the results were merged. This systematic review was registered in PROSPERO (CRD42022357570).

### 2.1 Quality assessment

The studies included in the final analysis were evaluated for risk of bias using Cochrane's Risk of Bias Tool for randomized controlled trials (Rob‐2) and the Joanna Briggs Institute's critical appraisal tools for cross‐sectional, quasi‐experimental, economic evaluation and qualitative studies [11, 12, 13, 14]. Three reviewers (YZ, SMG, JT) examined the risk of bias, and JJO resolved any discrepancies.

### 2.2 Data analysis

Statistical analyses were conducted in Stata 17 (StataCorp LP, College Station, USA), and a random‐effects model was used to estimate the pooled proportion of individuals linked to care or prevention post‐HIVST. Statistical heterogeneity was assessed using the *I* ^2^ statistic. For ease of analysis, studies that utilized multiple service delivery models were re‐categorized into a new “>1 type of service delivery model” sub‐category. Meta‐regression using backward elimination (including all variables with *p*< 0.2) was conducted to determine if any study‐level covariates could explain the between‐study heterogeneity. Doi plots were used to evaluate publication bias, and where available, time to linkage to care was presented in days.

### 2.3 Ethics

No ethical clearance was required by our institution to conduct the systematic review.

## 3 RESULTS

**Figure 1:**
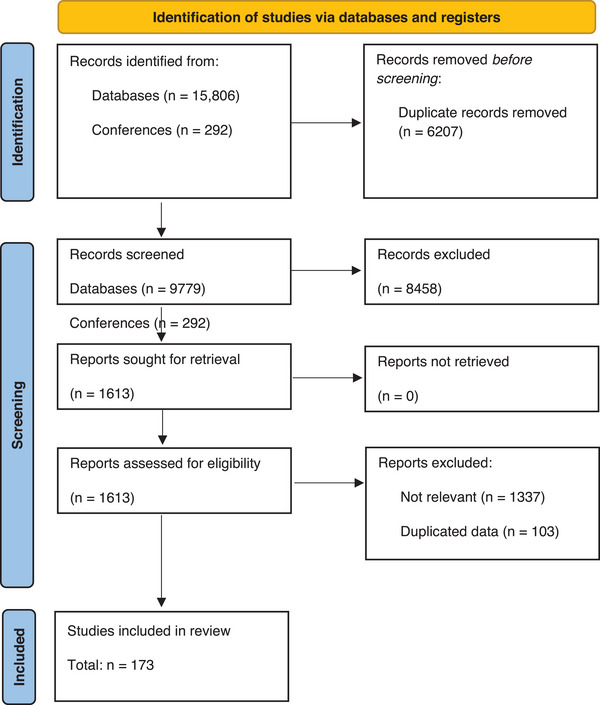
Prisma flow diagram.

In total, 10,071 studies were screened, of which 173 were included in our review (Figure 1 and Table S2). Most studies were non‐RCT and conducted in lower‐middle‐income countries in the African region using oral fluid‐based HIVST (Table 1). Studies that utilized assisted HIVST demonstrated a higher linkage to confirmatory testing, and initiation of ART in comparison to studies that opted for unassisted self‐test (Table 1 and Figures S1–S4). The mean time between enrolment and the linkage to any health services for participants reporting reactive HIVST results was 85 days (*n* = 48; SD = 145.3). High heterogeneity was observed in the pooled proportions in all the four categories; hence, a multivariable meta‐regression analysis was performed (Table 2).

**Table 1:** Summary of pooled proportions

	Number of studies	Proportion of people with reactive HIVST linked to confirmatory test (*N* = 124)	Number of studies	Proportion of people newly diagnosed with HIV linked to antiretroviral therapy (*N* = 88)	Number of studies	Proportion of people linked to HIV care (*N* = 69)	Number of studies	Proportion of people with non‐reactive HIVST linked to pre–exposure prophylaxis (*N* = 9)
**World region**								
African	52	0.89 (0.84–0.94)	49	0.85 (0.78–0.91)	30	0.67 (0.51–0.82)	2	0.01 (0.00–0.01)
Americas	14	0.88 (0.74–0.98)	8	0.78 (0.50–0.98)	13	0.98 (0.94–1.00)	2	0.05 (0.04–0.06)
Western Pacific	28	0.98 (0.92–1.00)	17	0.94 (0.88–0.98)	14	0.95 (0.80–1.00)	3	0.10 (0.02–0.23)
South‐East Asia	11	0.83 (0.62–0.98)	7	0.95 (0.86–1.00)	2	1.00 (1.00–1.00)	1	0.08 (0.03–0.16)
Eastern Mediterranean	3	1.00 (0.74–1.00)	2	1.00 (0.67–1.00)	1	1.00 (0.16–1.00)	0	Not applicable
European	11	0.95 (0.72–1.00)	2	1.00 (1.00–1.00)	2	0.92 (0.89–0.94)	1	0.00 (0.00–0.37)
**Country income level**								
High	24	0.86 (0.77–0.93)	11	0.85 (0.54–1.00)	13	0.98 (0.93–1.00)	3	0.15 (0.00–0.75)
Upper‐middle	41	0.91 (0.84–0.96)	25	0.91 (0.82–0.97)	24	0.83 (0.68–0.95)	3	0.10 (0.04–0.19)
Lower‐middle	39	0.93 (0.86–0.97)	27	0.93 (0.89–0.97)	14	0.84 (0.46–1.00)	3	0.04 (0.00–0.23)
Low	15	0.95 (0.88–0.99)	22	0.78 (0.66–0.88)	11	0.70 (0.57–0.81)	0	Not applicable
**Study design**								
Randomized controlled trial	16	0.82 (0.70–0.92)	17	0.74 (0.56–0.89)	19	0.74 (0.60–0.87)	1	0.05 (0.01–0.13)
Non‐trial	104	0.93 (0.90–0.96)	68	0.92 (0.89–0.95)	43	0.87 (0.77–0.96)	8	0.10 (0.02–0.21)
**Target population**								
Non‐key populations	45	0.87 (0.80–0.93)	31	0.87 (0.76–0.95)	21	0.70 (0.53–0.85)	3	0.26 (0.02–0.64)
Key populations	74	0.95 (0.91–0.98)	54	0.89 (0.84–0.93)	41	0.90 (0.80–0.97)	6	0.04 (0.00–0.12)
**Type of self‐testing kits**								
Oral‐fluid based	61	0.89 (0.83–0.94)	48	0.85 (0.76–0.92)	35	0.82 (0.68–0.94)	2	0.02 (0.01–0.03)
Fingerprick based	20	0.99 (0.95–1.00)	10	0.92 (0.81–1.00)	11	0.92 (0.81–0.99)	3	0.17 (0.00–0.68)
Both	8	0.91 (0.73–1.00)	6	1.00 (0.97–1.00)	3	0.63 (0.02–1.00)	0	Not applicable
Unclear	30	0.92 (0.84–0.98)	21	0.91 (0.85–0.96)	13	0.86 (0.65–0.99)	4	0.08 (0.00–0.22)
**Assisted versus unassisted**								
Unassisted	91	0.91 (0.86–0.95)	63	0.89 (0.83–0.95)	49	0.83 (0.71–0.93)	9	0.09 (0.02–0.19)
Assisted	16	0.98 (0.88–1.00)	15	0.91 (0.84–0.96)	7	1.00 (1.00–1.00)	0	Not applicable
Both	11	0.95 (0.86–1.00)	6	0.81 (0.60–0.95)	5	0.55 (0.14–0.93)	0	Not applicable
Unclear	1	0.82 (0.48–0.98)	1	0.44 (0.14–0.79)	1	1.00 (0.03–1.00)	0	Not applicable
**Service delivery model**								
Facility	18	0.96 (0.88–1.00)	11	0.91 (0.77–0.99)	13	0.91 (0.74–1.00)	2	0.07 (0.03–0.11)
Community (including community outreach, community counsellors)	24	0.92 (0.81–0.99)	20	0.85 (0.68–0.97)	7	0.46 (0.24–0.69)	0	Not applicable
Pharmacy	2	1.00 (0.98–1.00)	1	1.00 (0.91–1.00)	2	1.00 (1.00–1.00)	0	Not applicable
Online/mail	27	0.93 (0.81–1.00)	14	0.91 (0.79–0.99)	17	0.95 (0.84–1.00)	4	0.15 (0.02–0.37)
Social network‐based approach	19	0.99 (0.95–1.00)	13	0.90 (0.80–0.98)	7	0.88 (0.70–0.99)	0	Not applicable
Peer educator	11	0.97 (0.88–1.00)	13	0.86 (0.77–0.93)	6	0.77 (0.47–0.97)	1	0.01 (0.00–0.01)
Antenatal care‐delivered	5	0.59 (0.35–0.81)	6	0.92 (0.77–1.00)	3	0.60 (0.07–1.00)	0	Not applicable
Others (one‐stop shops, vending machine, schools)	2	0.65 (0.49–0.80)	2	0.34 (0.18–0.52)	3	0.47 (0.21–0.73)	0	Not applicable
>1 type of service delivery model	10	0.73 (0.56–0.86)	5	0.92 (0.83–1.00)	3	0.64 (0.18–0.98)	0	Not applicable
Unclear	19	1.00 (0.86–1.00)	0	NA	3	0.72 (0.08–1.00)	2	0.15 (0.13–0.16)
**Overall**		0.92 (0.89–0.96)		0.89 (0.84–0.93)		0.84 (0.74–0.93)		0.09 (0.02–0.19)

**Table 2:** Meta‐regression table

	Linkage to confirmatory testing (*N* = 124)				Linkage to antiretroviral therapy initiation (*N* = 88)				Linkage to care (*N* = 69)				Linkage to pre‐exposure prophylaxis (*N* = 9)			
	Crude odds ratio (95% CI)	*p*‐value	Adjusted odds ratio (95% CI)	*p*‐value	Crude odds ratio (95% CI)	*p*‐value	Adjusted odds ratio (95% CI)	*p*‐value	Crude odds ratio (95% CI)	*p*‐value	Adjusted odds ratio (95% CI)	*p*‐value	Crude odds ratio (95% CI)	*p*‐value	Adjusted odds ratio (95% CI)	*p*‐value
**World region**																
African	Reference		Reference		Reference		Reference		Reference		Reference		Reference		Reference	
Americas	0.96 (0.81–1.13)	0.593	0.96 (0.79–1.17)	0.663	0.94 (0.73–1.20)	0.622	1.00 (0.66–1.51)	0.997	1.32 (1.03–1.68)	0.026	1.43 (0.86–2.36)	0.158	1.33 (0.69–2.55)	0.293	Not applicable	Not applicable
Western Pacific	1.06 (0.96–1.17)	0.280	1.04 (0.92–1.17)	0.599	1.08 (0.95–1.22)	0.244	1.11 (0.91–1.35)	0.295	1.26 (1.03–1.55)	0.024	1.47 (1.00–2.17)	0.053	1.10 (0.61–1.99)	0.663	Not applicable	Not applicable
South‐East Asia	0.89 (0.78–1.03)	0.124	0.87 (0.74–1.00)	0.065	1.09 (0.89–1.35)	0.411	1.12 (0.88–1.43)	0.342	1.46 (0.94–2.28)	0.091	1.35 (0.64–2.87)	0.416	1.05 (0.46–2.42)	0.870	Not applicable	Not applicable
Eastern Mediterranean	1.17 (0.58–2.36)	0.656	1.12 (0.55–2.26)	0.770	1.22 (0.52–2.88)	0.641	0.95 (0.39–2.35)	0.918	1.46 (0.39–5.57)	0.569	1.77 (0.43–7.27)	0.413	Not applicable	Not applicable	Not applicable	Not applicable
European	1.03 (0.89–1.18)	0.707	0.99 (0.85–1.16)	0.802	1.18 (0.81–1.72)	0.391	1.18 (0.22–6.44)	0.845	1.33 (0.96–1.84)	0.087	1.16 (0.53–2.52)	0.700	0.97 (0.28–3.34)	0.954	Not applicable	Not applicable
**Country income level**																
High	Reference		Reference		Reference		Reference		Reference		Reference		Reference		Reference	
Upper‐middle	1.06 (0.91–1.23)	0.472	Not applicable	Not applicable	1.06 (0.85–1.33)	0.590	Not applicable	Not applicable	0.85 (0.64–1.12)	0.236	0.97 (0.59–1.59)	0.901	0.85 (0.05–1.34)	0.413	1.01 (0.72–1.40)	0.958
Lower‐middle	1.09 (0.94–1.27)	0.236	Not applicable	Not applicable	1.10 (0.88–1.37)	0.418	Not applicable	Not applicable	0.85 (0.63–1.15)	0.281	1.30 (0.75–2.26)	0.331	0.82 (0.53–1.28)	0.321	0.86 (0.65–1.15)	0.244
Low	1.11 (0.93–1.33)	0.249	Not applicable	Not applicable	0.94 (0.75–1.18)	0.599	Not applicable	Not applicable	0.78 (0.58–1.05)	0.101	1.17 (0.68–2.02)	0.561	Not applicable	Not applicable	Not applicable	Not applicable
**Study design**																
Randomized controlled trial	Reference		Reference		Reference		Reference		Reference		Reference		Reference		Reference	
Non‐trial	1.13 (0.99–1.30)	0.071	1.17 (1.01–1.35)	0.030	1.23 (1.10–1.38)	0.001	1.36 (1.15–1.61)	0.001	1.19 (1.01–1.42)	0.034	1.14 (0.86–1.53)	0.347	1.10 (0.62–1.95)	0.706	Not applicable	Not applicable
**Target population**																
Non‐key populations	Reference		Reference		Reference		Reference		Reference		Reference		Reference		Reference	
Key populations	1.05 (0.97–1.14)	0.238	1.04 (0.95–1.14)	0.348	1.01 (0.91–1.12)	0.854	1.20 (0.99–1.47)	0.069	1.20 (1.02–1.41)	0.026	0.97 (0.70–1.35)	0.850	0.74 (0.57–0.95)	0.027	0.72 (0.54–0.96)	0.031
**Type of HIVST kits**																
Oral‐fluid based	Reference		Reference		Reference		Reference		Reference		Reference		Reference		Reference	
Fingerprick based	1.09 (0.97–1.21)	0.146	Not applicable	Not applicable	1.05 (0.88–1.28)	0.548	0.81 (0.59–1.12)	0.191	0.76 (0.47–1.25)	0.276	0.96 (0.72–1.28)	0.782	Not applicable	Not applicable	Not applicable	Not applicable
Both	0.97 (0.82–1.13)	0.657	Not applicable	Not applicable	1.17 (0.90–1.51)	0.222	1.10 (0.85–1.41)	0.455	0.92 (0.71–1.17)	0.476	0.88 (0.49–1.57)	0.654	0.89 (0.59–1.35)	0.520	Not applicable	Not applicable
Unclear	Not applicable	Not applicable	Not applicable	Not applicable	Not applicable	Not applicable	Not applicable	Not applicable	Not applicable	Not applicable	Not applicable	Not applicable	Not applicable	Not applicable	Not applicable	Not applicable
**Assisted versus unassisted**																
Unassisted	Reference		Reference		Reference		Reference		Reference		Reference		Reference		Reference	
Assisted	1.05 (0.94–1.18)	0.386	1.01 (0.89–1.14)	0.941	1.04 (0.91–1.10)	0.496	1.02 (0.84–1.24)	0.807	1.29 (1.00–1.66)	0.050	1.32 (0.71–2.46)	0.359	Not applicable	Not applicable	Not applicable	Not applicable
Both	1.08 (0.95–1.22)	0.240	1.09 (0.96–1.25)	0.178	0.95 (0.81–1.12)	0.576	0.66 (0.51–0.85)	0.002	0.81 (0.63–1.04)	0.102	1.10 (0.74–1.65)	0.626	Not applicable	Not applicable	Not applicable	Not applicable
Unclear	Not applicable	Not applicable	Not applicable	Not applicable	Not applicable	Not applicable	Not applicable	Not applicable	Not applicable	Not applicable	Not applicable	Not applicable	Not applicable	Not applicable	Not applicable	Not applicable
**Service delivery model**																
>1 type of service delivery model	Reference		Reference		Reference		Reference		Reference		Reference		Reference		Reference	
Facility	1.17 (0.98–1.38)	0.071	1.23 (1.02–1.47)	0.030	0.94 (0.73–1.21)	0.623	0.90 (0.67–1.22)	0.494	1.11 (0.82–1.51)	0.498	1.23 (0.72–2.09)	0.436	Not applicable	Not applicable	Not applicable	Not applicable
Community (including community outreach, community counsellors)	1.13 (0.977–1.31)	0.097	1.15 (0.99–1.34)	0.074	0.87 (0.71–1.08)	0.205	0.96 (0.78–1.19)	0.708	0.72 (0.52–0.99)	0.042	0.81 (0.48–1.36)	0.419	Not applicable	Not applicable	Not applicable	Not applicable
Pharmacy	1.27 (0.87–1.85)	0.206	1.31 (0.89–1.92)	0.168	1.10 (0.67–1.80)	0.712	Not applicable	Not applicable	1.38 (0.84–2.27)	0.198	1.40 (0.34–5.67)	0.628	Not applicable	Not applicable	Not applicable	Not applicable
Online/mail	1.14 (0.98–1.32)	0.090	1.16 (0.99–1.36)	0.068	0.93 (0.74–1.18)	0.556	0.82 (0.59–1.14)	0.233	1.16 (0.87–1.55)	0.302	1.18 (0.71–1.96)	0.507	Not applicable	Not applicable	Not applicable	Not applicable
Social network‐based approach	1.27 (1.10–1.47)	0.002	1.28 (1.10–1.50)	0.001	0.94 (0.75–1.18)	0.586	0.89 (0.70–1.13)	0.344	1.21 (0.88–1.66)	0.232	1.26 (0.72–2.21)	0.404	Not applicable	Not applicable	Not applicable	Not applicable
Peer educator	1.21 (1.04–1.42)	0.018	1.17 (0.99–1.38)	0.073	0.91 (0.74–1.12)	0.375	0.78 (0.60–1.01)	0.062	1.05 (0.77–1.41)	0.762	1.29 (0.67–2.48)	0.437	Not applicable	Not applicable	Not applicable	Not applicable
Antenatal care‐delivered	0.98 (0.78–1.22)	0.832	1.02 (0.81–1.27)	0.903	0.99 (0.75–1.30)	0.918	1.12 (0.86–1.45)	0.403	0.90 (0.58–1.37)	0.609	1.05 (0.56–1.97)	0.864	Not applicable	Not applicable	Not applicable	Not applicable
Others (one‐stop shops, vending machine, schools)	0.93 (0.64–1.37)	0.717	0.93 (0.63–1.38)	0.708	0.57 (0.36–0.90)	0.017	0.48 (0.29–0.78)	0.004	0.81 (0.42–1.56)	0.522	0.84 (0.38–1.88)	0.668	Not applicable	Not applicable	Not applicable	Not applicable
Unclear	Not applicable	Not applicable	Not applicable	Not applicable	Not applicable	Not applicable	Not applicable	Not applicable	Not applicable	Not applicable	Not applicable	Not applicable	Not applicable	Not applicable	Not applicable	Not applicable
**Time to linkage (days)**	1.00 (1.00–1.00)	0.315	Not applicable	Not applicable	1.00 (1.00–1.00)	0.202	Not applicable	Not applicable	1.00 (1.00–1.00)	0.201	Not applicable	Not applicable	1.00 (0.98–1.01)	0.645	Not applicable	Not applicable

### 3.1 Linkage to confirmatory testing

**Figure 2:**
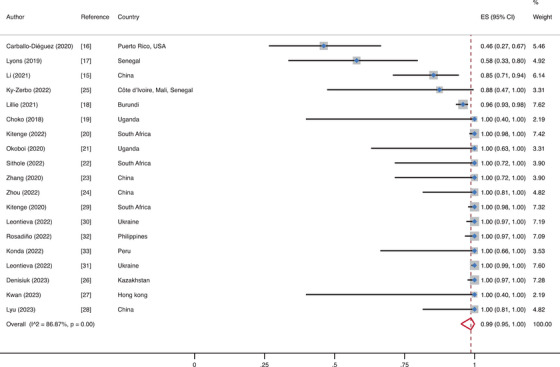
Forest plot for the proportion who were linked to confirmatory testing in social network‐based approach.

There were 124 studies that contained data on linkage to confirmatory test following a reactive HIVST result. Overall, 92% (95% CI: 88–95; *I* ^2^ = 96%) of individuals (*N* = 12,180/13,750) who tested reactive on HIVST sought confirmatory testing. Among the HIVST users in 19 studies using a social network‐based approach (SNA), 99% (95% CI: 95–100) were linked to confirmatory testing (Figure 2) [15, 16, 17, 18, 19, 20, 21, 22, 23, 24, 25, 26, 27, 28, 29, 30, 31, 32, 33]. Pooled proportions for other service delivery models can be found in Figures S5–S12. From our multivariable meta‐regression, we found that individuals who obtained HIVST kits through an SNA delivery model exhibited the highest odds (aOR = 1.28 [95% CI: 1.10–1.50], *p* = 0.001) of being linked to confirmatory testing, as compared to more than one type of service delivery model, after adjusting for world region, study design, population type and assistance during HIVST.

### 3.2 Linkage to ART initiation

Of the 173 studies, 88 studies (*N* = 8784/10,524 participants) reported on the number of individuals newly diagnosed with HIV who initiated ART. The overall pooled proportion of linkage to ART initiation was 89% (95% CI: 84–93; *I* ^2^ = 97%). Among studies that reported on ART initiation, 17 were RCTs and the remaining 68 were non‐RCT studies (Figures S13 and S14). The results of our meta‐regression analysis indicate that non‐RCTs exhibited a greater odds of ART initiation compared to RCTs (aOR = 1.36 [95% CI: 1.15–1.61], *p* = 0.001).

### 3.3 Linkage to care

There were 69 studies (*N* = 4133/7473 participants) that reported data on linkage to care following HIVST. Most studies provided definitions of care, which includes actions such as receiving a referral or attending a clinic, as well as being enrolled in a treatment facility. Several studies characterized care as being linked to prevention services such as counselling for behavioural risk reduction, and the provision of condoms and lubricants, or referrals for PrEP. The pooled proportion of “linkage to care” was 84% (95% CI: 74–93; *I* ^2^ = 98%).

### 3.4 Linkage to PrEP

Only nine studies (*N* = 351/8684 participants) reported data on linkage to PrEP (i.e. PrEP referral, PrEP initiation) for individuals who tested non‐reactive on HIVST, with a pooled proportion of 9% (95% CI: 2–19; *I* ^2^ = 99%). Of note, compared to 26% of individuals from the non‐key population, only 4% of those among the key population were linked to PrEP.

Using Doi plots, we detected minor to no publication bias in the studies included for their discussions of linkages to confirmatory testing (LFK index = −1.11), ART initiation (LFK index = −1.08) and PrEP (LFK index = −1.81). However, publication bias was detected in studies included for their discussion of linkages to care (LFK index = 5.95) (Supplementary Figures S15–S18). The quality assessment results are summarized in Tables S3–S6 and Figures S19 and S20. Of the RCT studies, all had some concerns due to deviations from intended interventions, and 89.4% (*n* = 42/47) of studies had some concerns with the outcome measurement (i.e. self‐reported HIVST results). A low risk of bias arose from the randomization process in 95.7% (*n* = 45/47) of studies, and all RCTs demonstrated a low risk of bias in the selection of reported results. Among the non‐trial studies, none presented nor clearly identified confounding factors, and none accounted for strategies to deal with them.

After removing studies with a high risk of bias, the results of the sensitivity analysis did not differ significantly. We found that individuals who acquired an HIVST kit through SNA were still more likely to be linked to confirmatory testing (aOR = 1.29 [95% CI: 1.08–1.54], *p* = 0.006) than those who acquired one from more than one type of service delivery model.

## 4 DISCUSSION

This systematic review explored linkages to care, including confirmatory testing and ART, and preventive measures, such as PrEP, following the use of an HIVST kit. In contrast to prior reviews that focused on specific geographical regions [34] or study designs [8], we consolidated the existing global evidence to offer insights that can guide the development of targeted interventions and strategies, ultimately strengthening the impact of HIVST programmes worldwide. The findings of this study show generally high levels of linkages to confirmatory testing (92%) and ART initiation (89%), albeit with significant differences regarding the service delivery model of the HIVST kit and the study design. Studies utilizing assisted HIVST demonstrated a higher linkage to confirmatory testing (98% [95% CI: 88–100]), ART initiation (91% [95% CI: 84–96]) and engagement in HIV care (100% [95% CI: 99–100]) compared to studies opting for unassisted self‐testing (91% [95% CI: 86–95], 89% [95% CI: 83–95], 83% [95% CI: 71–93], respectively). In addition, among the different service delivery model for HIVST kits, obtaining kits via social networks was found to have the highest odds of a later linkage to confirmatory testing post‐HIVST.

The prompt identification of people with reactive HIVST results and the subsequent initiation of ART for those diagnosed with HIV is essential for improving health outcomes and reducing ongoing transmission to sexual partners. While a confirmatory test following an HIVST is an essential step in the diagnostic process, it is not the only support that is needed following a reactive test. Individuals with a positive confirmatory test result need to undergo a thorough evaluation of their overall health and wellbeing by a clinician. Linkage to HIV care refers to the process of connecting individuals who have received an HIV diagnosis with appropriate medical care, support services and treatment. Individuals linked to care can access psychosocial support, counselling and education, as early access to medical care and support services is likely to improve the overall quality‐of‐life of PLHIV.

Therefore, it is crucial to recognize the potential factors associated with higher linkages to care post‐HIVST. Identifying these facilitators can inform the implementation and expansion of programmes and shape policy development. Our results suggest that using SNA to distribute HIVST kits is positively associated with linkages to care post‐HIVST. Furthermore, in 2023, the WHO recommended SNA‐based HIV testing in order for all populations to increase testing coverage and strengthen the uptake of HIV prevention and treatment services [35]. SNA leverages the relationships and connections within a community to disseminate information, resources or interventions. In the context of HIV testing, SNA involves utilizing individuals’ existing social ties to encourage and facilitate testing behaviours among peers [36]. By tapping into trusted social connections, this approach enhances the reach of HIVST kits and improves linkages to care. It recognizes that individuals are influenced by their social environment, where norms, behaviours and attitudes are shaped by interactions with family, friends and peers. Prior research has demonstrated that the health‐seeking behaviours of individuals can be influenced by members of their social networks, as these behaviours are intricately woven into the dynamic social structures or networks that connect individuals to one another through various interactions. For example, in a study of SNA in South Africa, 9891 individuals distributed 31,341 HIVST kits (median of three kits per peer); among those with reactive HIVST results, 100% (196/196) proceeded to undergo confirmatory testing [20]. Furthermore, a process evaluation of the peer‐to‐peer delivery of HIVST among youth in rural KwaZulu‐Natal, South Africa, found that the peer‐to‐peer approach was well received and that the participants were comfortable sharing sexual health issues that they would not have otherwise disclosed to adults [37]. In addition, strategies for secondary distribution by companions, partners or peers could leverage existing sexual and social networks to reach people who would not otherwise test for HIV, potentially linking them to care [27, 38, 39, 40, 41].

In addition to linking people with reactive HIVST results to confirmatory testing and ART initiation, linking those with non‐reactive HIVST results to preventive care is of equal significance. This linkage provides an opportunity for education regarding safer practices and prevention methods, especially the initiation of PrEP. However, in our study, we found that only nine of the papers (*N* = 351/8684) reported some form of linkage to PrEP for participants with negative HIVST results, of which most studies were aimed at measuring linkage to ART. One strategy to increase the likelihood of successful linkage is to embed PrEP services within HIVST programmes. By integrating PrEP into HIVST, individuals who test negative for HIV would be able to immediately receive information and access to preventive measures, thus strengthening the overall prevention framework. Implementing educational resources (e.g. PrEP, safe sex) and navigation services post self‐testing can aid to overcome some of the potential barriers and facilitate a smooth transition to PrEP initiation. These barriers often include lack of awareness about PrEP, limited access to healthcare providers and stigmatization associated with HIV prevention, particularly in marginalized communities. Additionally, logistical hurdles such as transportation, high costs and the complexity of insurance coverage can further prevent individuals from starting PrEP. By addressing these obstacles and providing clear guidance, additional emphasis could be placed on streamlining the HIV testing requirements and prescription process, thus mitigating the systemic barriers that impede PrEP initiation. Furthermore, HIVST has the potential to facilitate greater differentiated service delivery for PrEP initiation and continuation in new settings beyond the bounds of traditional healthcare facilities, such as in private pharmacies or during at‐home visits [42]. PrEP services can be integrated into community settings to enhance accessibility, particularly for individuals in remote or underserved areas, reducing the need for frequent facility visits. HIVST can also be used to reassure PrEP users that their prevention practices are effective, thereby encouraging them to continue their regimen. Future research should explore HIVST‐supported models of PrEP delivery, the role of community‐based organizations and the integration of digital health technologies, to ensure that individuals who self‐test for HIV are seamlessly linked to PrEP as a vital component of the global HIV prevention strategy.

Our findings should be interpreted in light of several important limitations. First, high heterogeneity was observed among the studies included in the sample and we explored heterogeneity using meta‐regression. A further explanation of the observed heterogeneity could be our inclusion of a broad range of evidence from multiple settings delivering HIVST to various populations through a multitude of intervention types. In settings with a higher background prevalence of HIV, there may be more people who test positive through HIVST, creating a greater need for linkage to treatment or prevention services like PrEP. Furthermore, different populations, such as MSM, sex workers or people in rural areas, may have varied barriers to accessing care, such as stigma, discrimination or logistical challenges like transportation. Additionally, age, sex, gender and socio‐economic status can influence awareness of HIV prevention options, healthcare access and willingness to engage with the healthcare system after a self‐test. For example, younger individuals or those from marginalized communities may be less likely to seek out follow‐up services due to fear of stigma or lack of healthcare literacy. This underlines the importance of developing interventions based on the needs of the local setting and target populations. Second, other factors could have influenced linkage to care that were not captured, for example, background prevalence of HIV, differences in time to linkage, differences in population demographics and study design variations. In addition, every study was observed for varying durations; thus, studies with longer observational periods may have exhibited a greater likelihood of more individuals being linked to care. Some studies had on‐site confirmatory testing, potentially resulting in higher linkage rates. In our study, although the non‐RCT studies reported greater odds of ART initiation compared to the RCT studies, it is possible that the RCT studies were not designed to encourage or measure linkage. Another reason could be a greater proportion of key‐population focused (e.g. MSM and transgender people) non‐RCTs compared to RCTs. There could be a greater loss to follow‐up among non‐RCT studies, resulting in an artificially high linkage to care among those who reported data. Furthermore, we also detected a high risk of bias due to the inherent nature of the way in which the data were captured for HIVST, whereby individuals provided with HIVST kits self‐reported their testing behaviour to researchers, and the HIVST trials were not blinded. We acknowledge the possibility that some individuals with reactive tests would not have reported their results or sought medical care. Finally, we were unable to disaggregate the outcomes by sex, as the majority of studies did not specifically report the gender of participants who were linked to confirmatory testing, ART initiation or PrEP. Future research should aim to report linkage outcomes stratified by sex and gender.

## 5 CONCLUSIONS

In conclusion, this systematic review contributes to the ongoing discourse surrounding HIVST by shedding light on the critical step of linking individuals to appropriate care and treatment post‐HIVST. High linkage to confirmatory testing and ART initiation after HIVST was reported, particularly with types of assisted HIVST and the use of SNA. Future research needs to prioritize understanding the use of HIVST for PrEP, beyond linkage and start including initiation and continuation. Timely linkage is crucial to improving health outcomes, ending HIV transmission and reaching the UNAIDS 95‐95‐95 goal, and ongoing research and collaboration with community‐based organizations are needed to address the challenges and ensure positive health outcomes for individuals with positive HIVST results.

## COMPETING INTERESTS

All authors declare no competing interests.

## AUTHORS’ CONTRIBUTIONS

YZ and JJO conceived the idea for this paper. YZ, SMG and JT did the screening and data extraction. YZ conducted the statistical analysis. All authors had full access to all the data in the study contributed to the interpretation and subsequent edits of the manuscript and had final responsibility for the decision to submit for publication.

## Supporting information




**Table S1**. Literature search strategy
**Table S2**. Summary of included studies
**Table S3**. Quality assessment of qualitative studies
**Table S4**. Quality assessment of randomised controlled trial studies
**Table S5**. Quality assessment of quasi‐experimental studies
**Table S6**. Quality assessment for cross‐sectional studies
**Figure S1**. Forest plot for the proportion who were linked to confirmatory testing in unassisted HIVST
**Figure S2**. Forest plot for proportion who were linked to confirmatory testing in assisted HIVST
**Figure S3**. Forest plot for proportion who were linked to ART initiation reports in unassisted HIVST
**Figure S4**. Forest plot for proportion who were linked to ART initiation reports in assisted HIVST
**Figure S5**. Forest plot for proportion who were linked to confirmatory testing from health facility delivery model
**Figure S6**. Forest plot for proportion who were linked to confirmatory testing from community delivery model
**Figure S7**. Forest plot for proportion who were linked to confirmatory testing from pharmacy delivery model
**Figure S8**. Forest plot for proportion who were linked to confirmatory testing from online/mail delivery model
**Figure S9**. Forest plot for proportion who were linked to confirmatory testing from peer‐educator delivery model
**Figure S10**. Forest plot for proportion who were linked to confirmatory testing from antenatal care delivery model
**Figure S11**. Forest plot for proportion who were linked to confirmatory testing from other delivery models
**Figure S12**. Forest plot for proportion who were linked to confirmatory testing with >1 type of delivery models
**Figure S13**. Forest plot for proportion who were linked to ART initiation reports in RCT
**Figure S14**. Forest plot for proportion who were linked to ART initiation reports in non‐RCT
**Figure S15**. Doi plot of included studies for linkage to confirmatory testing
**Figure S16**. Doi plot of included studies for linkage to ART initiation
**Figure S17**. Doi plot of included studies for linkage to care
**Figure S18**. Doi plot of included studies of linkage to PrEP
**Figure S19**. Summary graph for risk of bias of RCT studies
**Figure S20**. Visual graph for risk of bias of RCT studies

## Data Availability

Data will be made available upon request made to the corresponding author.
