# Supplementary material for: Linkage to care and prevention after HIV self‐testing: a systematic review and meta‐analysis
Source: J Int AIDS Soc. 2024 Dec 11;27(12):e26388. doi: 10.1002/jia2.26388 (PMC11634545; doi:10.1002/jia2.26388)

#### Supplementary

Table of Contents

[Table S1. Literature search strategy 3](#_Toc177155882)

[Table S2. Summary of included studies 4](#_Toc177155883)

[Figure S1. Forest plot for the proportion who were linked to confirmatory testing in unassisted HIVST 23](#_Toc177155884)

[Figure S2. Forest plot for proportion who were linked to confirmatory testing in assisted HIVST 24](#_Toc177155885)

[Figure S3. Forest plot for proportion who were linked to ART initiation reports in unassisted HIVST 25](#_Toc177155886)

[Figure S4. Forest plot for proportion who were linked to ART initiation reports in assisted HIVST 26](#_Toc177155887)

[Figure S5. Forest plot for proportion who were linked to confirmatory testing from health facility delivery model 27](#_Toc177155888)

[Figure S6. Forest plot for proportion who were linked to confirmatory testing from community delivery model 28](#_Toc177155889)

[Figure S7. Forest plot for proportion who were linked to confirmatory testing from pharmacy delivery model 29](#_Toc177155890)

[Figure S8. Forest plot for proportion who were linked to confirmatory testing from online/mail delivery model 30](#_Toc177155891)

[Figure S9. Forest plot for proportion who were linked to confirmatory testing from peer-educator delivery model 31](#_Toc177155892)

[Figure S10. Forest plot for proportion who were linked to confirmatory testing from antenatal care delivery model 32](#_Toc177155893)

[Figure S11. Forest plot for proportion who were linked to confirmatory testing from other delivery models 33](#_Toc177155894)

[Figure S12. Forest plot for proportion who were linked to confirmatory testing with >1 type of delivery models 34](#_Toc177155895)

[Figure S13. Forest plot for proportion who were linked to ART initiation reports in RCT 35](#_Toc177155896)

[Figure S14. Forest plot for proportion who were linked to ART initiation reports in non-RCT 36](#_Toc177155897)

[Figure S15. Doi plot of included studies for linkage to confirmatory testing 37](#_Toc177155898)

[Figure S16. Doi plot of included studies for linkage to ART initiation 37](#_Toc177155899)

[Figure S17. Doi plot of included studies for linkage to care 38](#_Toc177155900)

[Figure S18. Doi plot of included studies of linkage to PrEP 38](#_Toc177155901)

[Table S3. Quality assessment of qualitative studies 39](#_Toc177155902)

[Table S4. Quality assessment of randomised controlled trial studies 41](#_Toc177155903)

[Table S5. Quality assessment of quasi-experimental studies 45](#_Toc177155904)

[Table S6. Quality assessment for cross-sectional studies 46](#_Toc177155905)

[Figure S19. Summary graph for risk of bias of RCT studies 66](#_Toc177155906)

[Figure S20. Visual graph for risk of bias of RCT studies 67](#_Toc177155907)

Table S1. Literature search strategy

| Search Number | Search strategy |
| --- | --- |
| 1 | (“HIV infection*” or “ HIV” or “HIV1” or “HIV2” or “HIV type 1” or “HIV type 2” or “human immunodeficiency virus” or “human immun* adj3 deficiency virus” or “acquired immunodeficiency syndrome” or “acquired immun* adj3 deficiency syndrome” or “Sexually transmitted diseases, Viral”).mp |
| 2 | (“Diagnostic Self Evaluation” or “Self-Testing” or “home test*” or “rapid test*” or “home self test*” or “home-based self test” or “self test*” or “HIV self-test*” or “HIVST”).mp |
| 3 | exp HIV Testing/ and (home* or self* or rapid*).mp |
| 4 | 1 and 2 |
| 5 | 3 or 4 |

## Table S2. Summary of included studies

| **Author** | **Year of Publication** | **Title** | **Intervention** | **Assisted vs unassisted** | **Country** | **World region** | **Country income level** | **Type of HIVST** | **Study Design** | **Population type** | **Service delivery model** | **Time to linkage (days/weeks/months)** |
| --- | --- | --- | --- | --- | --- | --- | --- | --- | --- | --- | --- | --- |
| Adepoju et al. | 2022 | How efficient are HIV self-testing models? A comparison of community, facility, one-stop-shop and pharmacy retail distribution models in Nigeria | 4 HIVST distribution models targeting men, key populations, adolescents, young people and orphans and vulnerable children | Unassisted | Nigeria | African | Lower Middle | Oral-fluid | Non-RCT | Non-key population | Community, Facility, One-stop shop, Pharmacy |  |
| Altaf et al. | 2022 | Acceptability and feasibility of HIV self-testing among transgender people in Larkana, Pakistan: Results from a pilot project | Oral fluid HIVST distribution by trained transgender peer outreach workers | Unassisted | Pakistan | Eastern Mediterranean | Lower Middle | Oral-fluid | Non-RCT | Key populations and sexual partners of pregnant mothers | Community | 5 working days |
| Altaf, et al. | 2023 | Acceptability and feasibility of intra-peer and social media approach for HIV self-testing among men who have sex with men in Karachi: lessons from a pilot study | A community-based organisation conducted a peer-to-peer and social media approach to distribute HIV self-testing kits to men who have sex with men. | Unassisted | Pakistan | Eastern Mediterranean | Lower Middle | Oral-fluid | Non-RCT | MSM | Community |  |
| Anand et al. | 2017 | Implementation of an online HIV prevention and treatment cascade in Thai men who have sex with men and transgender women using Adam's Love Electronic Health Record system | E-counselling for HIV and enrolment into HIVST and counselling | Assisted | Thailand | South-East Asian | Upper Middle | Fingerprick | Non-RCT | MSM | Community |  |
| Bbuye et al. | 2022 | Factors Associated with Linkage to HIV Care Among Oral Self-Tested HIV Positive Adults in Uganda | Quantitative interviews of self-tested HIV positive participants | Both | Uganda | African | Low | Oral-fluid | Non-RCT | Non-key population | Facility, Community |  |
| Bell et al. | 2021 | Online HIV self-testing (HIVST) dissemination by an Australian community peer HIV organisation: A scalable way to increase access to testing, particularly for suboptimal testers | A free online HIVST dissemination service hosted by a peer-led, community-based organisation with on-site, peer-facilitated HIV testing and referral programs | Unassisted | Australia | Western Pacific | High | Oral-fluid | Non-RCT | Non-key population | Online/mail | <2 weeks |
| Belza et al. | 2012 | Supervised blood-based self-sample collection and rapid test performance: a valuable alternative to the use of saliva by HIV testing programmes with no medical or nursing staff | Street-based HIV testing programme | Assisted | Spain | European | High | Fingerprick | Non-RCT | Non-key population | Community |  |
| Bil et al. | 2019 | Usage of purchased self-Tests for HIV infections among migrants living in the UK, France and the Netherlands: A cross-sectional study | Logistic regression analyses of web-based questionnaire data on HIVST usage | Unassisted | United Kingdom, France, Netherlands | European | High | Unclear | Non-RCT | Non-key population | Unclear |  |
| Boni et al. | 2019 | An Internet-Based HIV Self-Testing Program to Increase HIV Testing Uptake Among Men Who Have Sex With Men in Brazil: Descriptive Cross-Sectional Analysis | A free, anonymous, internet-based HIVST project under the “A Hora é Agora” (The Time is Now) program | Unassisted | Brazil | the Americas | Upper Middle | Oral-fluid | Non-RCT | MSM | Online/mail |  |
| Brady et al. | 2017 | HIV self-testing: Feasibility and acceptability of a large scale national service | Pilot of a national HIVST service using a dedicated website and promoting the service on social media | Unassisted | United Kingdom | European | High | Unclear | Non-RCT | MSM | Online/mail |  |
| Carballo-Diéguez et al. | 2020 | Use of Rapid HIV Self-Test to Screen Potential Sexual Partners: Results of the ISUM Study | Use of rapid HIV self-test to screen potential sexual partners | Unassisted | Puerto Rico, America | the Americas | High | Oral-fluid | RCT | MSM, Transgender people | SNA |  |
| Cary et al. | 2017 | HIV self-testing: feasibility and acceptability of a large-scale national service delivered by a community organization | HIV self-test website | Unassisted | United Kingdom | European | High | Unclear | Non-RCT | MSM | Online/mail |  |
| Cassell et al. | 2022 | A cross-sectional assessment of HIV self-testing preferences and uptake among key populations in Phnom Penh, Cambodia | HIVST or referrals to facility-based testing offered through community outreach or via online advertising | Both | Cambodia | Western Pacific | Lower Middle | Both | Non-RCT | MSM, Transgender people | Community | Within 3 months (ART linkage) |
| Chan et al. | 2021 | Effectiveness of a novel HIV self-testing service with online real-time counseling support (HIVST-online) in increasing HIV testing rate and repeated HIV testing among men who have sex with men in Hong Kong: Results of a pilot implementation project | HIVST with online real-time counselling intervention | Assisted | Hong Kong | Western Pacific | High | Oral-fluid | Non-RCT | MSM | Online/mail |  |
| Chanda et al. | 2017 | HIV self-testing among female sex workers in Zambia: a randomized controlled trial | Trial of direct HIVST distribution, HIVST coupon as HIVST distribution mechanisms conducted by trained peer educators | Unassisted | Zambia | African | Low | Oral-fluid | RCT | FSW | Facility |  |
| Chesang et al. | 2016 | Using peer educators to scale-up HIV oral self-testing among female sex workers: An implementation science approach from Kenya | Qualitative interviews to determine acceptance, competence, feasibility of FSW peer educators-led HIVST distribution | Unassisted | Kenya | African | Lower Middle | Oral-fluid | Non-RCT | FSW | Peer educator | >6 months |
| Choko et al. | 2019 | HIV self-testing alone or with additional interventions, including financial incentives, and linkage to care or prevention among male partners of antenatal care clinic attendees in Malawi: An adaptive multi-arm, multi-stage cluster randomised trial | Male partners of antenatal care clinic attendees | Unassisted | Malawi | African | Low | Oral-fluid | RCT | Partners of KP | ANC-delivered | 28 days |
| Choko et al. | 2021 | Partner-delivered HIV self-test kits with and without financial incentives in antenatal care and index patients with HIV in Malawi: a three-arm, cluster-randomised controlled trial | Trial involving the use of enhanced soc, HIVST only and HIVST plus financial incentive arms targeting male partner of antenatal care patients | Unassisted | Malawi | African | Low | Oral-fluid | RCT | Partners of KP | ANC-delivered | 28 days |
| Choko et al. | 2015 | Uptake, Accuracy, Safety, and Linkage into Care over Two Years of Promoting Annual Self-Testing for HIV in Blantyre, Malawi: A Community-Based Prospective Study | HIVST offered by trained resident volunteer-counsellors to adult residents | Unassisted | Malawi | African | Low | Oral-fluid | RCT | Non-key population | Facility |  |
| Choko et al. | 2018 | A pilot trial of the peer-based distribution of HIV self-test kits among fishermen in Bulisa, Uganda | Pilot trial of the secondary distribution of HIVST through peer networks | Unassisted | Uganda | African | Low | Oral-fluid | Non-RCT | Non-key population | SNA |  |
| Conserve, et al. | 2023 | Peer-based promotion and nurse-led distribution of HIV self-testing among networks of men in Dar es salaam, Tanzania: Development and feasibility results of the STEP Intervention | Peer-based promotion and nurse-led distribution of HIV self-testing | Unassisted | Tanzania | African | Lower Middle | Oral-fluid | Non-RCT | Non-key population | Community | 1 month |
| da Cruz et al. | 2021 | Comprehensive approach to HIV/AIDS testing and linkage to treatment among men who have sex with men in Curitiba, Brazil | Secondary analysis of self-tested MSM | Unassisted | Brazil | the Americas | Upper Middle | Oral-fluid | Non-RCT | MSM | Online/mail | less than 90 days (average 51 days) |
| De Boni et al. | 2018 | Self-testing, communication and information technology to promote HIV diagnosis among young gay and other men who have sex with men (MSM) in Brazil | Analysis of programmatic data collected for each of A Hora é Agora’s HTC strategies | Unassisted | Brazil | the Americas | Upper Middle | Oral-fluid | Non-RCT | MSM | Online/mail |  |
| De Schacht et al. | 2022 | Reaching Men and Young Adults in a Pharmacy-Based HIV Self-Testing Strategy: Results from an Acceptability Study in Mozambique | Pilot comprehensive HIVST services implemented at government-owned free-standing or private pharmacies | Unassisted | Mozambique | African | Low | Oral-fluid | RCT | Non-key population | Pharmacy |  |
| Denisiuk, et al. | 2023 | Social Network Strategy improves access to HIV testing and harm reduction programs for PWID and their partners in Kazakhstan | Secondary analysis of the data collected during the Optimized HIV case finding | Assisted | Kazakhstan | European | Upper Middle | Oral-fluid | Non-RCT | PWID | SNA |  |
| DiCarlo et al. | 2020 | HIV self-testing reaches previously unreached key and vulnerable populations in Democratic Republic of the Congo (DRC) | Peer educators trained to offer HIVST to key populations | Assisted | Democratic Republic of the Congo | African | Low | Oral-fluid | Non-RCT | FSW, MSM, and FSW clients | Peer educator |  |
| Dovel et al. | 2020 | Effect of facility-based HIV self-testing on uptake of testing among outpatients in Malawi: a cluster-randomised trial | An index HIVST intervention among ART clients, involving clients distribution of HIVST to primary sexual partners | Assisted | Malawi | African | Low | Oral-fluid | RCT | Non-key population | Facility | 3 months |
| Dovel, et al. | 2023 | Effect of index HIV self-testing for sexual partners of clients enrolled in antiretroviral therapy (ART) programs in Malawi: A randomized controlled trial | HIVST intervention integrated into high-burden outpatient departments | Unassisted | Malawi | African | Low | Oral-fluid | RCT | Partners of KP | SNA | 12 months |
| Edelstein et al. | 2020 | Five Waves of an Online HIV Self-Test Giveaway in New York City, 2015 to 2018 | An online home test giveaway in which codes were emailed to redeem a free HIVST online | Unassisted | America | the Americas | High | Oral-fluid | Non-RCT | MSM, Transgender people | Online/mail | 2 months |
| Eustaquio et al. | 2022 | Outcomes of a community-led online-based HIV self-testing demonstration among cisgender men who have sex with men and transgender women in the Philippines during the COVID-19 pandemic: a retrospective cohort study | A community-led online-based HIVST demonstration | Unassisted | Philippines | Western Pacific | Lower Middle | Fingerprick | Non-RCT | MSM, Transgender people | Online/mail | 12 weeks |
| Fernandez et al. | 2022 | High linkage to treatment among key populations who self-test through a peer HIV self-test distribution and community-based ART program among key populations in Lagos, Nigeria | Employing trusted peers to reach key populations with HIVST | Unassisted | Nigeria | African | Lower Middle | Unclear | Non-RCT | MSM | Peer educator |  |
| Floyd et al. | 2018 | Linkage to HIV care following HIV self-testing: a cluster randomised trial of community-based distribution of oral HIV self-test kits nested in four HPTN 071 communities in Zambia | HIVST cluster randomised trial nested within HPTN 071 study | Both | Zambia | African | Low | Oral-fluid | RCT | Non-key population | Community | 3 months |
| Gashobotse et al. | 2019 | Peer-mobilized HIV self-testing increases case detection and linkage to ART among key populations in Burundi | Peer-mobilized HIV self-testing | Assisted | Burundi | African | Low | Unclear | Non-RCT | MSM, FSW | Peer educator |  |
| Girault et al. | 2021 | Uptake of oral fluid-based HIV self-testing among men who have sex with men and transgender women in Thailand | HIVST or referral to HIV testing services offered during outreach and online activities | Both | Thailand | South-East Asian | Upper Middle | Oral-fluid | Non-RCT | MSM, Transgender people | Community |  |
| Greacen et al. | 2012 | Access to and use of unauthorised online HIV self-tests by internet-using French-speaking men who have sex with men | Analysis of online questionnaire to identify factors associated with accessing HIVST | Unassisted | France | European | High | Unclear | Non-RCT | Non-key population | Online/mail |  |
| Green et al. | 2018 | From conventional to disruptive: upturning the HIV testing status quo among men who have sex with men in Vietnam | HIV lay provider and HIVST strategies piloted alongside intensive social media outreach | Unassisted | Vietnam | Western Pacific | Lower Middle | Fingerprick | Non-RCT | MSM | Community |  |
| Guadamuz et al. | 2022 | A pilot feasibility study of HIV self-testing with online supervision among MSM who attend sexualized drug parties in Thailand | Online supervised HIVST for men who attend sexualised drug parties | Assisted | Thailand | South-East Asian | Upper Middle | Fingerprick | Non-RCT | MSM | Online/mail |  |
| Hacking et al. | 2022 | HIV Self-testing Among Previously Diagnosed HIV-Positive People in Khayelitsha, South Africa: No Evidence of Harm but may Facilitate Re-engagement in ART Care | HIVST distributed at a private pharmacy and at HIV testing outreach events | Unassisted | South Africa | African | Upper Middle | Oral-fluid | Non-RCT | Non-key population | Pharmacy, Community | 6 months? |
| Hector et al. | 2018 | Acceptability and performance of a directly assisted oral HIV self-testing intervention in adolescents in rural Mozambique | A directly assisted oral HIVST intervention | Assisted | Mozambique | African | Low | Oral-fluid | Non-RCT | Adolescents | Facility | immediately |
| Herath et al. | 2022 | Launching oral-based HIV self-testing (HIVST) in Sri Lanka through the COVID-19 pandemic | HIVST distribution for the National STD/AIDS Control Program | Unassisted | Sri Lanka | South-East Asian | Lower Middle | Oral-fluid | RCT | Non-key population | Mixed |  |
| Horvath et al. | 2021 | Histep: A single-arm pilot study of a technology-assisted hiv self-testing intervention in kampala, uganda | Pilot HIVST intervention called histep which involves text messages, live support and “last-mile” HIVST kit delivery | Unassisted | Uganda | African | Low | Oral-fluid | Non-RCT | Non-key population | Online/mail |  |
| Huang et al. | 2016 | Using Grindr, a Smartphone Social-Networking Application, to Increase HIV Self-Testing Among Black and Latino Men Who Have Sex With Men in Los Angeles, 2014 | Social networking app used for HIVST promotion | Unassisted | America | the Americas | High | Oral-fluid | Non-RCT | MSM | Online/mail |  |
| Huang et al. | 2022 | Towards the first 90: impact of the national HIV self-test program on case finding and factors associated with linkage to confirmatory diagnosis in Taiwan | Quasi-experimental interrupted time-series (ITS) analysis, and an analysis of a retrospective cohort of PLWHIV with initial positive results on hivsts | Unassisted | Taiwan | Western Pacific | High | Oral-fluid | Non-RCT | Non-key population | Facility, Vending machine, Online | 1 week to 123 weeks |
| Hubbard et al. | 2022 | Improving ART initiation among men who use HIV self-testing in Malawi: a qualitative study | In-depth interviews with men who tested HIV positive using HIVST | Unassisted | Malawi | African | Low | Unclear | Non-RCT | Non-key population | Facility | 2 weeks |
| Indravudh et al. | 2021 | Effect of community-led delivery of HIV self-testing on HIV testing and antiretroviral therapy initiation in Malawi: A cluster-randomised trial | Mobilisation of community health groups to lead 7-day HIVST campaigns | Unassisted | Malawi | African | Low | Oral-fluid | RCT | Non-key population | Community |  |
| Jamil et al. | 2017 | Effect of availability of HIV self-testing on HIV testing frequency in gay and bisexual men at high risk of infection (FORTH): a waiting-list randomised controlled trial | Randomised trial assessing the effect of availability of free HIV self-testing on the frequency of HIV testing in two groups of high-risk gay and bisexual men (FORTH trial) | Unassisted | Australia | Western Pacific | High | Oral-fluid | RCT | MSM | Facility |  |
| Jin et al. | 2019 | An internet-based self-testing Model (Easy Test): Cross-sectional survey targeting men who have sex with men who never tested for HIV in 14 provinces of China | Feasibility study of the Easy Test model through which individuals can order free HIVST kits after submitting a US$5 deposit | Unassisted | China | Western Pacific | Upper Middle | Fingerprick | Non-RCT | MSM | Online/mail |  |
| Johnson et al. | 2022 | Combating Stigma Through HIV Self-Testing: New York State's HIV Home Test Giveaway Program for Sexual Minorities | Coupon provided via email for a free HIVST | Unassisted | America | the Americas | High | Oral-fluid | Non-RCT | MSM, Transgender people | Online/mail |  |
| Joseph Davey et al. | 2022 | HIV testing and linkage to ART following secondary distribution of HIV self-test kits to male partners of women living with HIV: a pilot randomized control trial in Mpumalanga, South Africa | Secondary distribution of HIV self-test kits to male partners of women living with HIV | Unassisted | South Africa | African | Upper Middle | Oral-fluid | RCT | Partners of KP | Facility | 3 months |
| Junior Matovu et al. | 2022 | Acceptability, willingness to use and preferred distribution models of oral-based HIV self-testing kits among key and priority populations enrolled in HIV pre-exposure prophylaxis clinics in central Uganda. A mixed-methods cross-sectional study | Key populations and priority populations provided HIVST demonstration and option to do HIVST | Unassisted | Uganda | African | Low | Oral-fluid | Non-RCT | MSM,People who inject drugs, FSW and priority populations (Fisherfolks, Adolescent girls and young women and negative people in discordant sexual relationships) | Facility |  |
| Katz et al. | 2018 | HIV Self-Testing Increases HIV Testing Frequency in High-Risk Men Who Have Sex With Men: a Randomized Controlled Trial | High-risk HIV-negative MSM provided access to HIVST at no cost | Unassisted | America | the Americas | High | Oral-fluid | RCT | MSM, Transgender people | Facility | 15 months (study period) |
| Khajehkazemi et al. | 2021 | Feasibility of HIV self-testing among female sex workers in Iran: the SELFii study | Analysis of national bio-behavioural surveillance surveys | Both | Iran | Eastern Mediterranean | Lower Middle | Unclear | Non-RCT | FSW | Peer educator |  |
| Khanal et al. | 2020 | From a demonstration study to national rollout: HIV self-testing in Nepal | Key population community driven demonstration study | Unassisted | Nepal | South-East Asian | Lower Middle | Unclear | RCT | MSM, Transgender people | Community |  |
| Kikuli, et al. | 2023 | Peer client bond in accelerating the return of unassisted HIV self-test kits among female sex workers. Afya jumuishi Geita region | Peer-client bonding in accelerating the return of unassisted HIV self-test kits among female sex workers | Unassisted | Tanzania | African | Lower Middle | Unclear | Non-RCT | FSW | Peer educator |  |
| Kimaru et al. | 2018 | HIV self-test distribution by young women: an innovative way to reach men in rural South Africa | Young women given invitations for clinic-based HIV counselling and testing or HIVST to use and share with peers and male partners | Unassisted | South Africa | African | Upper Middle | Oral-fluid | RCT | Adolescents | SNA |  |
| Kitenge et al. | 2022 | Acceptability of unsupervised peer-based distribution of HIV oral self-testing for the hard-to-reach in rural KwaZulu Natal, South Africa: Results from a demonstration study | Lay counsellors or community health workers provided HIVST kits to primary recipients for distribution to sexual partners | Unassisted | South Africa | African | Upper Middle | Oral-fluid | Non-RCT | Non-key population | SNA | 2 months |
| Kitenge et al. | 2020 | Unassisted peer-based distribution of HIV oral self-testing for the hard to reach in rural Kwazulu Natal, South Africa | Peer distribution of HIVST | Unassisted | South Africa | African | Upper Middle | Oral-fluid | RCT | Non-key population | SNA |  |
| Konda et al. | 2022 | HIV self-testing to increase combination prevention demand among men who have sex with men (MSM) and transgender women (TGW): a randomized clinical trial and sub-study of the ImPrEP project | HIVST secondary distribution by PrEP users | Unassisted | Brazil, Peru | the Americas | Upper Middle | Unclear | RCT | MSM | SNA |  |
| Koris et al. | 2021 | Youth-friendly HIV self-testing: Acceptability of campus-based oral HIV self-testing among young adult students in Zimbabwe | Campus-based HIVST using lay workers for distribution | Unassisted | Zimbabwe | African | Lower Middle | Oral-fluid | Non-RCT | Non-key population | Community |  |
| Korte et al. | 2020 | HIV Oral Self-Testing for Male Partners of Women Attending Antenatal Care in Central Uganda: uptake of Testing and Linkage to Care in a Randomized Trial | HIV oral self-testing for male partners of women attending antenatal care | Unassisted | Uganda | African | Low | Oral-fluid | Non-RCT | Partners of KP | ANC-delivered |  |
| Kra, et al. | 2023 | HIV self-testing positivity rate and linkage to confirmatory testing and care: a telephone survey in Cote d'Ivoire, Mali and Senegal | Distribution of HIVST among key populations, including female sex workers and men who have sex with men, and secondary distribution of HIVST to their partners, peers and clients. (ALTAS program) | Unassisted | Côte d’Ivoire, Mali, Senegal | African | Lower Middle | Both | Non-RCT | MSM, FSW | Community, Facility | 3 months |
| Kumwenda et al. | 2019 | Post-test adverse psychological effects and coping mechanisms amongst HIV self-tested individuals living in couples in urban Blantyre, Malawi | Interviews with HIV self-tested individuals living in couples | Unassisted | Malawi | African | Low | Oral-fluid | Non-RCT | Non-key population | Community |  |
| Kwan, et al. | 2023 | Implementation Cascade of a Social Network-Based HIV Self-testing Approach for Men Who Have Sex with Men: Cross-sectional Study | Implementation of social network–based HIV self-test approach for reaching MSM | Unassisted | Hong Kong | Western Pacific | High | Both | Non-RCT | MSM | SNA |  |
| Ky-Zerbo, et al. | 2022 | I take it and give it to my partners who will give it to their partners: Secondary distribution of HIV self-tests by key populations in Cote d'Ivoire, Mali, and Senegal | Secondary distribution of HIV self-tests by key populations | Unassisted | Côte d’Ivoire, Mali, Senegal | African | Lower Middle | Oral-fluid | Non-RCT | MSM, FSW | SNA |  |
| Larsson, et al. | 2023 | Feasibility and usability of mobile technology to assist HIV self-testing in youth in Zimbabwe: A mixed-methods study | Mobile application to assist HIV self-testing | Unassisted | Zimbabwe | African | Lower Middle | Oral-fluid | Non-RCT | Adolescents | Facility |  |
| Lavoie et al. | 2022 | Scale-up and optimization of HIV self-testing for the last mile in Botswana | Implementing HIVST across 53 facilities in Botswana | Assisted | Botswana | African | Upper Middle | Unclear | Non-RCT | Non-key population | Facility | three days |
| Lebina et al. | 2019 | Feasibility of community-based HIV self-screening in South Africa: a demonstration project | HIV self-screening through mobile site, home-based, workplace and sex worker programmes | Both | South Africa | African | Upper Middle | Oral-fluid | Non-RCT | Non-key population | Community |  |
| Leontieva et al. | 2022 | Advancing peer-driven social network testing (SNT) and index testing to maximize reach of HIV testing services (HTS) among men who have sex with men (MSM) and their contacts in Ukraine | Peer-driven social network testing and expanded index testing of MSM and their contacts | Unassisted | Ukraine | European | Lower Middle | Unclear | Non-RCT | MSM | SNA |  |
| Leontieva et al. | 2022 | Pragmatically approaching social network testing (SNT): using a peer-driven community outreach model to extend reach of HIV testing services (HTS) to networks of people who inject drugs (PWID) in Ukraine | Former or soon-to-be released PWID prisoners as seed recruiters to mobilize social, sexual, and drug injecting contacts for HIVST | Unassisted | Ukraine | European | Lower Middle | Unclear | Non-RCT | Non-key population | SNA |  |
| Li et al. | 2021 | Feasibility of Indirect Secondary Distribution of HIV Self-test Kits via WeChat Among Men Who Have Sex With Men: National Cross-sectional Study in China | Indirect secondary distribution of HIVST kits via WeChat | Unassisted | China | Western Pacific | Upper Middle | Fingerprick | Non-RCT | MSM | SNA |  |
| Lillie et al. | 2021 | Increasing Testing Options for Key Populations in Burundi Through Peer-Assisted HIV Self-Testing: Descriptive Analysis of Routine Programmatic Data | Peer-assisted HIVST provided by existing peer outreach workers | Assisted | Burundi | African | Low | Oral-fluid | RCT | MSM, FSW, Transgender people | Peer educator |  |
| Lippman et al. | 2018 | Ability to use oral fluid and fingerstick HIV self-testing (HIVST) among South African MSM | Usability of oral-fluid and fingerstick HIV self-testing kits among South African MSM | Assisted | South Africa | African | Upper Middle | Both | Non-RCT | MSM | Facility |  |
| Lippman et al. | 2018 | High Acceptability and Increased HIV-Testing Frequency After Introduction of HIV Self-Testing and Network Distribution Among South African MSM | HIVST provided for use and for network distribution | Unassisted | South Africa | African | Upper Middle | Both | RCT | MSM | SNA |  |
| Lyons et al. | 2019 | Use and Acceptability of HIV Self-Testing Among First-Time Testers at Risk for HIV in Senegal | HIVST kits distributed through targeted venues | Unassisted | Senegal | African | Lower Middle | Oral-fluid | Non-RCT | Non-key population | SNA |  |
| Lyu, et al. | 2023 | Factors associated with first-time HIV testing among MSM via secondary distribution of HIV self-test kits in Zhuhai, China | Secondary distribution of HIV self‐test kits | Unassisted | China | Western Pacific | Upper Middle | Unclear | Non-RCT | MSM | SNA |  |
| Maatouk et al. | 2021 | Community-led HIV self-testing for men who have sex with men in Lebanon: lessons learned and impact of COVID-19 | Workshops conducted to introduce HIVST services for healthcare workers working at different NGOs | Unassisted | Lebanon | Eastern Mediterranean | Lower Middle | Oral-fluid | Non-RCT | MSM | Community |  |
| MacGowan et al. | 2020 | Effect of Internet-Distributed HIV Self-tests on HIV Diagnosis and Behavioral Outcomes in Men Who Have Sex With Men: A Randomized Clinical Trial | To evaluate the effect of providing HIV self-tests on frequency of testing, diagnoses of HIV infection, and sexual risk behaviors. (estamp trial) | Unassisted | USA | the Americas | High | Oral-fluid | RCT | MSM | Online/mail |  |
| MacPherson et al. | 2014 | Effect of optional home initiation of HIV care following HIV self-testing on antiretroviral therapy initiation among adults in Malawi: a randomized clinical trial | Positively tested participants who used HIVST allocated to optional home initiation of HIV care or to facility-based care | Unassisted | Malawi | African | Low | Oral-fluid | RCT | Non-key population | Community |  |
| Majam et al. | 2019 | High self-reporting of HIV self-test results through an interactive voice response telephone line in inner city Johannesburg | Use of short message service reminders and interactive voice response hotline to aid reporting of HIVST use and linkage to care | Unassisted | South Africa | African | Upper Middle | Unclear | Non-RCT | Non-key population | Online/mail |  |
| Majam, et al. | 2022 | Incentives to promote accessing HIV care and viral suppression among HIV self-screening test users who obtain a reactive result | RCT to examine if reminders and financial rewards have an effect on engagement in care | Unassisted | South Africa | African | Upper Middle | Unclear | RCT | Adolescents | Facility |  |
| Marlin et al. | 2014 | Piloting an HIV self-test kit voucher program to raise serostatus awareness of high-risk African Americans, Los Angeles | A voucher program for free HIVST kits targeting African American MSM | Unassisted | America | the Americas | High | Oral-fluid | Non-RCT | MSM | Community |  |
| Masters et al. | 2016 | Promoting Partner Testing and Couples Testing through Secondary Distribution of HIV Self-Tests: A Randomized Clinical Trial | HIVST kits provided to mothers and couples testing through secondary distribution of HIVST | Unassisted | Kenya | African | Lower Middle | Oral-fluid | RCT | Non-key population | ANC-delivered |  |
| Matovu et al. | 2020 | Feasibility and acceptability of a pilot, peer-led HIV self-testing intervention in a hyperendemic fishing community in rural Uganda | HIVST kits distribution by trained peer-leaders from existing social networks | Unassisted | Uganda | African | Low | Oral-fluid | Non-RCT | Non-key population | Peer educator | within 1 week of HIV diagnosis |
| Matsuda, et al. | 2022 | Impact of covid-19 on people living with HIV-1: care and prevention indicators at a local and nationwide level, Santo Andre, Brazil | Secondary analysis of data from the Brazilian Ministry of Health official registry of HIV/AIDS care and prevention indicators | Unclear | Brazil | the Americas | Upper Middle | Unclear | Non-RCT | Non-key population | Facility |  |
| Mavedzenge et al. | 2017 | Feasibility of HIV self-test programming among female sex workers in Zimbabwe | HIVST in female sex workers in Zimbabwe | Unassisted | Zimbabwe | African | Lower Middle | Unclear | Non-RCT | FSW | Facility | 2 weeks |
| Mavhu et al. | 2021 | Innovative demand creation strategies to increase voluntary medical male circumcision uptake: a pragmatic randomised controlled trial in Zimbabwe | Voluntary medical male circumcision demand creation augmented by human-centred design-informed approach | Unassisted | Zimbabwe | African | Lower Middle | Unclear | RCT | Non-key population | Facility |  |
| McHugh, et al. | 2023 | On campus HIV self-testing distribution at tertiary level colleges in Zimbabwe increases access to HIV testing for youth | On campus HIV self-testing distribution | Unassisted | Zimbabwe | African | Lower Middle | Oral-fluid | Non-RCT | Adolescents | Others | 1 month |
| Medley et al. | 2019 | Outcome and cost of 3 methods for increasing male partner testing in South Africa | Different HIV testing options offered to pregnant women whose partner was HIV negative or unknown | Unassisted | South Africa | African | Upper Middle | Oral-fluid | Non-RCT | Non-key population | ANC-delivered |  |
| Mee et al. | 2021 | Factors associated with the successful distribution of HIV self-tests through routine clinical settings in Lusaka, Zambia: a cohort study | Secondary distribution of HIV self-tests in individuals accessing antenatal care to their partners | Unassisted | Zambia | African | Low | Unclear | Non-RCT | Antenatal/pregnancy | ANC-delivered |  |
| Mkopi, et al. | 2023 | Acceptability and uptake of oral HIV self-testing among rural community members in Tanzania: a pilot study | Oral HIV self-testing among rural community members | Unassisted | Tanzania | African | Lower Middle | Oral-fluid | Non-RCT | Non-key population | Community | 1 month |
| Moore et al. | 2019 | Investigating the addition of oral HIV self-tests among populations with high testing coverage - Do they add value? Lessons from a study in Khayelitsha, South Africa | HIVST kits provided to participants who reported their results by pre-paid SMS or by returning to facility | Unassisted | South Africa | African | Upper Middle | Oral-fluid | Non-RCT | Non-key population | Community | 6 months and >6months |
| Moore et al. | 2017 | Self-testing: an effective means of increasing HIV-testing and status awareness | Pilot study of HIVST at two health facilities | Unassisted | South Africa | African | Upper Middle | Unclear | Non-RCT | Non-key population | Facility |  |
| Mshweshwe-Pakela et al. | 2022 | Digitally supported HIV self-testing increases facility-based HIV testing capacity in Ekurhuleni, South Africa | Integrating digitally supported HIVST into the clinic | Assisted | South Africa | African | Upper Middle | Oral-fluid | Non-RCT | Non-key population | Facility | 14 days; 9 months |
| Muchedzi et al. | 2021 | High HIV Positivity Rates Following Large-Scale HIV Self-Testing Implementation in Zimbabwe, 2018-2020 | Analysis of HIVST data from the Zimbabwe HIV Care and Treatment project | Both | Zimbabwe | African | Lower Middle | Oral-fluid | Non-RCT | Non-key population | Community |  |
| Mugo et al. | 2017 | Uptake and acceptability of oral HIV self-testing among community pharmacy clients in Kenya: A feasibility study | HIVST kits offered at community pharmacies for purchase | Unassisted | Kenya | African | Lower Middle | Oral-fluid | Non-RCT | Non-key population | Pharmacy |  |
| Mulubwa et al. | 2019 | Community based distribution of oral HIV self-testing kits in Zambia: a cluster-randomised trial nested in four HPTN 071 (PopART) intervention communities | Cluster-randomised study nested in the HPTN 071 (PopART) trial | Unassisted | Zambia | African | Low | Oral-fluid | RCT | Non-key population | Community |  |
| Mutale et al. | 2021 | Addition of HIV self-test kits to partner notification services to increase HIV testing of male partners of pregnant women in Zambia: two parallel randomised trials | Use of partner notification services and targeted education for pregnant women on HIVST kit use for their partners | Unassisted | Zambia | African | Low | Oral-fluid | RCT | Non-key population | ANC-delivered |  |
| Mwanda et al. | 2021 | Extending reach of HIV testing services (HTS) through private-sector outlets: feasibility of offering HIV self-testing (HIVST) at pharmacies and alternative medicine centers (AMC) in Democratic Republic of the Congo (DRC) | Tested a public-private model for HIVST in pharmacy | Assisted | Democratic Republic of the Congo | African | Low | Unclear | Non-RCT | Non-key population | Pharmacy |  |
| Nangendo, et al. | 2023 | Village health team-delivered oral HIV self-testing increases linkage-to-care and antiretroviral-therapy initiation among men in Uganda | Village health team-delivered oral HIV self-testing | Unassisted | Uganda | African | Low | Oral-fluid | Non-RCT | Non-key population | Community | 3 months to ART |
| Napierala et al. | 2019 | HIV self-testing services for female sex workers, Malawi and Zimbabwe | Evaluation of sex workers’ preferences for test kit distribution , and a process evaluation | Unassisted | Malawi; Zimbabwe | African | Low; Lower Middle | Oral-fluid | Non-RCT | FSW | SNA | within 2-4 weeks |
| Nasuuna et al. | 2022 | Early observations from the HIV self-testing program among key populations and sexual partners of pregnant mothers in Kampala, Uganda: a cross sectional study | Peer-to-peer model for key populations and secondary distribution for partners of consenting pregnant and lactating mothers | Unassisted | Uganda | African | Low | Oral-fluid | Non-RCT | key populations and sexual partners of pregnant motherS | Peer educator |  |
| Neuman et al. | 2021 | Does community-based distribution of HIV self-tests increase uptake of HIV testing? Results of pair-matched cluster randomised trial in Zambia | HIVST kits distributed by community-based distributors | Unassisted | Zambia | African | Low | Oral-fluid | RCT | Non-key population | Community |  |
| Nguyen et al. | 2022 | HIV self-testing reached the our key populations regardless of COVID-19 | Web-based HIV self-test distribution | Unassisted | Vietnam | Western Pacific | Lower Middle | Unclear | Non-RCT | Non-key population | Online/mail |  |
| Nguyen et al. | 2019 | Community-led HIV testing services including HIV self-testing and assisted partner notification services in Vietnam: lessons from a pilot study in a concentrated epidemic setting | HIV testing services offered at drop-in houses or coffee shops, which were assisted and observed by peer educators | Unassisted | Vietnam | Western Pacific | Lower Middle | Oral-fluid | Non-RCT | MSM, key populations | Peer educator |  |
| Ngwalle, et al. | 2023 | Successful return of client's results after an HIV self-test: The role of peer educators in southern highlands, Tanzania | Peer educations impact on return of HIV self-testing results | Both | Tanzania | African | Lower Middle | Unclear | Non-RCT | Non-key population | Peer educator |  |
| Nwafor et al. | 2022 | HIVST among children, adolescents and pregnant and breastfeeding women: implementation results and enablers for scale-up | Secondary distribution of HIVST by adolescent champions to their peers | Unassisted | Nigeria | African | Lower Middle | Unclear | Non-RCT | Adolescents | Peer educator |  |
| O'Byrne et al. | 2021 | At-home HIV self-testing during COVID: implementing the GetaKit project in Ottawa | The GetaKit project for at-home HIVST for HIV negative and non-prep users | Unassisted | Canada | the Americas | High | Fingerprick | Non-RCT | Non-key population | Online/mail |  |
| Okoboi et al. | 2020 | Peer distribution of HIV self-test kits to men who have sex with men to identify undiagnosed HIV infection in Uganda: A pilot study | Secondary distribution of HIVST through MSM peer networks at the AIDS Support Organisation centres | Unassisted | Uganda | African | Low | Oral-fluid | Non-RCT | MSM | SNA |  |
| Oldenburg et al. | 2018 | Brief Report: intimate Partner Violence and Antiretroviral Therapy Initiation Among Female Sex Workers Newly Diagnosed With HIV in Zambia: a Prospective Study | HIVST distribution among FSW by peer educators | Unassisted | Zambia | African | Low | Unclear | RCT | FSW | Peer educator |  |
| Ortblad et al. | 2017 | Direct provision versus facility collection of HIV self-tests among female sex workers in Uganda: A cluster-randomized controlled health systems trial | Direction HIVST provision and provision of coupon for free HIVST collection in a healthcare facility | Unassisted | Uganda | African | Low | Oral-fluid | RCT | FSW | Peer educator | 1-4 months |
| Owusu et al. | 2020 | Acceptability of provider assisted self-testing among men who have sex with men in Ghana | Assisted HIVST among MSM by a civil society organization in Ghana | Assisted | Ghana | African | Lower Middle | Unclear | Non-RCT | MSM | Community |  |
| Pai et al. | 2013 | Will an unsupervised self-testing strategy for HIV work in health care workers of South Africa? A cross sectional pilot feasibility study | Point-of-care HIV test, HIVST applications and mobile phones were synergised for use to create an unsupervised HIVST strategy | Unassisted | South Africa | African | Upper Middle | Oral-fluid | Non-RCT | Non-key population | Facility | 8 hrs |
| Pai et al. | 2018 | An Unsupervised Smart App-Optimized HIV Self-Testing Program in Montreal, Canada: Cross-Sectional Study | App-optimised HIVST strategy, with the hivsmart! App mimicking an unsupervised home environment | Unassisted | Canada | the Americas | High | Oral-fluid | Non-RCT | MSM | Facility | within the same day of test |
| Pai et al. | 2021 | Impact of a personalised, digital, HIV self-testing app-based program on linkages and new infections in the township populations of South Africa | Offsite unsupervised and on-site supervised digital HIVST program with 24/7 linkage services | Unassisted | South Africa | African | Upper Middle | Oral-fluid | RCT | Non-key population | Community |  |
| Pasipamire et al. | 2020 | Implementation of community and facility-based HIV self-testing under routine conditions in southern Eswatini | Assisted and unassisted HIVST offered at HIV testing services sites | Unassisted | Eswatini | African | Lower Middle | Oral-fluid | Non-RCT | Non-key population | Facility, Community |  |
| Phanuphak et al. | 2020 | Linkages to HIV confirmatory testing and antiretroviral therapy after online, supervised, HIV self-testing among Thai men who have sex with men and transgender women | Online, supervised HIVST among Thai MSM and TGW | Assisted | Thailand | South-East Asian | Upper Middle | Fingerprick | Non-RCT | MSM, Transgender people | Online/mail |  |
| Phatsoane Gaven, et al. | 2023 | HIV self-test reporting using mHealth platforms: A pilot study in Johannesburg, South Africa | HIV self-testers reporting their results using a mobile-health (mhealth) platform | Unassisted | South Africa | African | Upper Middle | Fingerprick | Non-RCT | Non-key population | Online/mail |  |
| Phongphiew et al. | 2021 | Acceptability of blood-based HIV self-testing among adolescents aged 15–19 years at risk of HIV acquisition in Bangkok | Acceptability of HIVST study among at-risk adolescents | Unassisted | Thailand | South-East Asian | Upper Middle | Fingerprick | Non-RCT | MSM | Facility | on the same day |
| Pisemskiy | 2018 | Effective promotion of HIV Self-testing among MSM in Russia in the context of growing stigma and discrimination | Free HIVST kits for MSM | Unassisted | Russia | European | Upper Middle | Unclear | Non-RCT | MSM, Transgender people | Facility, Pharmacy, Clubs, Sauna |  |
| Pollard et al. | 2022 | Tailoring HIVST to local communities can improve uptake: lessons learned from a virtual HIV self-testing intervention in India | Web-based platform for HIVST with virtual counsellors | Assisted | India | South-East Asian | Lower Middle | Unclear | Non-RCT | Non-key population | Online/mail |  |
| Qin et al. | 2016 | Benefits and adverse outcomes of HIV self-testing among high-risk MSM in China: an implementation perspective | A nationwide online survey among MSM in China | Unassisted | China | Western Pacific | Upper Middle | Both | Non-RCT | MSM | Unclear |  |
| Ren et al. | 2017 | HIV care-seeking behaviour after HIV self-testing among men who have sex with men in Beijing, China: A cross-sectional study | An online survey among users of a gay networking app in China | Unassisted | China | Western Pacific | Upper Middle | Unclear | Non-RCT | MSM | Unclear |  |
| Robson et al. | 2020 | A community-based intervention (men's spaces) to engage men in hiv and sexual health services in Malawi: a pilot study | A community-based intervention to engage men to discuss their sexual health | Unassisted | USA | the Americas | High | Unclear | Non-RCT | Non-key population | Community | same day |
| Rodger | 2022 | Free HIV self-test for identification and linkage to care of previously undetected HIV infection in men who have sex with men in England and Wales (SELPHI): an open-label, internet-based, randomised controlled trial | internet-based, open-label, randomised controlled trial among MSM to receive free HIVST (SELPHI study) | Unassisted | UK | European | High | Fingerprick | RCT | MSM, Transgender people | Online/mail | 3 months |
| Rosadiño et al. | 2022 | SelfCare's Cat and Pao: the use of community-led automated virtual assistance and peer support in an unassisted HIV self-testing program in Metro Manila, Philippines | Virtual assistance and peer support in a HIV self-testing program | Assisted | Philippines | Western Pacific | Lower Middle | Unclear | Non-RCT | Non-key population | Online/mail |  |
| Rosengren et al. | 2016 | Feasibility of using GrindrTM to distribute HIV self-test kits to men who have sex with men in Los Angeles, California | Free HIVST kits advertised on a gay dating app targeting a high-risk HIV population | Unassisted | USA | the Americas | High | Fingerprick | Non-RCT | MSM | Online/mail |  |
| Rosenthal, et al. | 2023 | Geospatial Prioritization to Reach Hispanic or Latino and Other Priority Populations Through HIV Home Testing Services | New York City HIV Home Test Giveaway for key populations | Unassisted | United States | the Americas | High | Unclear | Non-RCT | Non-key population | Online/mail | Up to 11 weeks |
| Salvadori, et al. | 2022 | Uptake, acceptability and interpretability of 3-in-1 rapid blood self-testing for HIV, hepatitis B and hepatitis C | Use of a 3-in-1 self-test for HIV, Hepatitis B & C. | Unassisted | Thailand | South-East Asian | Upper Middle | Fingerprick | Non-RCT | Non-key population, MSM | Facility |  |
| Sarkar et al. | 2016 | Feasibility of supervised self-testing using an oral fluid-based HIV rapid testing method: a cross-sectional, mixed method study among pregnant women in rural India | HIVST instructions provided and self-testing under supervision of a community health worker conducted | Assisted | India | South-East Asian | Lower Middle | Oral-fluid | Non-RCT | Antenatal/pregnancy | Community |  |
| Sayabounthavong et al. | 2019 | Optimizing individual and public health benefits of assisted HIV self-testing through effective linkages to services for men who have sex with men and transgender women in Laos | Secondary analysis of the data collected in the LINKAGES project | Unassisted | Laos | Western Pacific | Lower Middle | Unclear | Non-RCT | MSM, Transgender people | Community |  |
| Shahmanesh et al. | 2019 | Mafrica: Zenzele, a mobile-phone enabled HIV testing and linkage to care pathway for young people in rural South Africa | Formative study for co-developing and piloting Zenele mobile phone app for HIVST | Both | South Africa | African | Upper Middle | Unclear | Non-RCT | Adolescents | Online/mail |  |
| Shally et al. | 2020 | Lessons learned from transgender women peers offering HIV-self testing and assisted partner notification services in malindi, KENYA | Transgender women peers offering HIV-self testing | Assisted | Kenya | African | Lower Middle | Oral-fluid | Non-RCT | Transgender people | Peer educator |  |
| Sibanda et al. | 2021 | Comparison of community-led distribution of HIV self-tests kits with distribution by paid distributors: a cluster randomised trial in rural Zimbabwean communities | Trial of community-led distribution of HIVSTK and HIVSTK distribution by paid distributors | Unassisted | Zimbabwe | African | Lower Middle | Oral-fluid | RCT | Non-key population | Community |  |
| Sibanda et al. | 2021 | Community-based HIV self-testing: a cluster-randomised trial of supply-side financial incentives and time-trend analysis of linkage to antiretroviral therapy in Zimbabwe | HIVST distributed door-to-door by community-based distributors | Unassisted | Zimbabwe | African | Lower Middle | Oral-fluid | RCT | Non-key population | Community |  |
| Sithole et al. | 2022 | Secondary Distribution of HIV Self-Testing Kits to Social and Sexual Networks of PLWH in KwaZulu-Natal, South Africa. A Brief Report | PLWHIV as HIVST kit distributors for secondary distribution of HIVST kits | Unassisted | South Africa | African | Upper Middle | Both | Non-RCT | Social and network contacts of PLHIV | SNA |  |
| Sithole et al. | 2021 | Implementation of HIV self-testing to reach men in rural uMkhanyakude, KwaZulu-Natal, South Africa. a DO-ART trial sub study | HIVST kits distributed following community sensitisation | Unassisted | South Africa | African | Upper Middle | Both | Non-RCT | Non-key population | Community | 7.4 months |
| Stafylis et al. | 2018 | Vending machines in commercial sex venues to increase HIV self-testing among men who have sex with men | Free HIVST kits distributed using vending machines at commercial sex venues | Unassisted | USA | the Americas | High | Oral-fluid | Non-RCT | MSM | Others |  |
| Stafylis, et al. | 2022 | Relative Effectiveness of Social Media, Dating Apps, and Information Search Sites in Promoting HIV Self-testing: Observational Cohort Study | To compare the relative effectiveness of social media sites, dating apps, and information search sites in promoting HIV self-testing among MSM | Unclear | United States | the Americas | High | Oral-fluid | Non-RCT | Non-key population | Online/mail |  |
| Stephenson et al. | 2022 | Efficacy of a Telehealth Delivered Couples' HIV Counseling and Testing (CHTC) Intervention to Improve Formation and Adherence to Safer Sexual Agreements Among Male Couples in the US: Results from a Randomized Control Trial | A telehealth delivering couples’ HIV counselling and testing intervention | Assisted | USA | the Americas | High | Oral-fluid | RCT | MSM | Online/mail | 30 days |
| Stephenson et al. | 2020 | Project Moxie: Results of a Feasibility Study of a Telehealth Intervention to Increase HIV Testing Among Binary and Nonbinary Transgender Youth | HIVST kit via mail and testing conducted under supervision of a remotely-located counsellor during a prescheduled video-chat session | Assisted | USA | the Americas | High | Oral-fluid | RCT | Transgender | Online/mail | linked to care within 30 days |
| Tama et al. | 2021 | Does onsite HIV self-testing enhance linkage to care? A case study of HIVST Challenge Fund Project in Kenya | Trained young women as peer educators to distribute HIVST | Unassisted | Kenya | African | Lower Middle | Unclear | Non-RCT | Non-key population | Peer educator |  |
| Tang et al. | 2019 | What happens after HIV self-testing? Results from a longitudinal cohort of Chinese men who have sex with men | Secondary analysis of data of an intervention trial to increase HIV testing | Unassisted | China | Western Pacific | Upper Middle | Unclear | Non-RCT | MSM | Online/mail | confirmed results at facility-based sites within the same 3-month follow up period |
| Tao et al. | 2014 | Home-based HIV testing for men who have sex with men in China: A novel community-based partnership to complement government programs | HIV rapid testing kits mailed | Unassisted | China | Western Pacific | Upper Middle | Fingerprick | Non-RCT | MSM | Online/mail |  |
| Thirumurthy et al. | 2016 | Promoting male partner HIV testing and safer sexual decision making through secondary distribution of self-tests by HIV-negative female sex workers and women receiving antenatal and post-partum care in Kenya: a cohort study | Secondary distribution of HIVST by HIV-uninfected FSW and women receiving antenatal and postpartum care | Unassisted | Kenya | African | Lower Middle | Oral-fluid | Non-RCT | FSW | SNA |  |
| Tonen-Wolyec et al. | 2019 | Acceptability, feasibility, and individual preferences of blood-based HIV self-testing in a population-based sample of adolescents in Kisangani, Democratic Republic of the Congo | A peer-based supervised HIVST conducted in a door-to-door survey | Assisted | DRC | African | Low | Fingerprick | Non-RCT | Non-key population | Peer educator |  |
| Tonen-Wolyec et al. | 2020 | Comparison of practicability and effectiveness between unassisted HIV self-testing and directly assisted HIV self-testing in the Democratic Republic of the Congo: a randomized feasibility trial | Unassisted and directly assisted HIVST in the field setting | Both | DRC | African | Low | Fingerprick | RCT | Non-key population | Facility |  |
| Tonen-Wolyec et al. | 2021 | Uptake of HIV/AIDS Services Following a Positive Self-Test Is Lower in Men Than Women in the Democratic Republic of the Congo | A secondary analysis of data from surveys to explore linkage to care for HIVST | Both | DRC | African | Low | Fingerprick | Non-RCT | Non-key population | Facility | 30 days, mean 9.7+/- 2.4 days |
| Trabwongwitaya, et al. | 2022 | Preference and ability to perform blood-versus oral-fluid-based HIV self-testing in adolescents and young adults in Bangkok | Adolescents and young adult participants between 18 and 24 years-old to perform HIV self-test | Unassisted | Thailand | South-East Asian | Upper Middle | Both | Non-RCT | Adolescents | Facility |  |
| Tukei et al. | 2019 | HIV self-test uptake, yield, and linkage experiences among key populations in LESOTHO | HIVST kits distributed by trained peer educators and HIV testing service counsellors | Unassisted | Lesotho | African | Lower Middle | Unclear | Non-RCT | MSM, Transgender people | Peer educator |  |
| Tumusiime et al. | 2022 | Peer-led community HIV self-testing (HIVST) distribution models improves access to HIV testing services (HTS) among key populations (KP) in Uganda | Peer-led community HIV self-testing distribution | Both | Uganda | African | Low | Unclear | Non-RCT | Key populations | Peer educator |  |
| Tun et al. | 2018 | Uptake of HIV self-testing and linkage to treatment among men who have sex with men (MSM) in Nigeria: A pilot programme using key opinion leaders to reach MSM | HIVST kit distribution conducted at a baseline survey | Unassisted | Nigeria | African | Lower Middle | Oral-fluid | Non-RCT | MSM | Peer educator |  |
| Udeagu et al. | 2017 | Men Who Have Sex With Men Seek Timely Human Immunodeficiency Virus Confirmation and Care After Rapid Human Immunodeficiency Virus Self-Test: Data From Partner Services Program, New York City | Analysis of data from HIV partner services program | Unassisted | USA | the Americas | High | Oral-fluid | Non-RCT | Non-key population | Facility | 3 months |
| Vera et al. | 2019 | Acceptability and feasibility of using digital vending machines to deliver HIV self-Tests to men who have sex with men | Developing digital vending machines to deliver HIVST | Unassisted | UK | European | High | Fingerprick | Non-RCT | MSM | Vending machine |  |
| Villares et al. | 2021 | User assessment of HIV self-testing (HIVST) in Brazil: An acceptable tool with great potential for reaching key populations and maximize positivity yield | An online self-administered structured questionnaire to assess user acceptance and experience using HIVST | Both | Brazil | the Americas | Upper Middle | Unclear | Non-RCT | MSM, key populations | Pharmacy, SNA, Facility |  |
| Volk et al. | 2016 | Acceptability and feasibility of HIV self-testing among men who have sex with men in Peru and Brazil | Participants trained to use HIVST and instructed to use the self-test monthly | Unassisted | Brazil, Peru | the Americas | Upper Middle; Upper Middle | Fingerprick | Non-RCT | MSM | Facility | 3 months |
| Vu et al. | 2020 | Leading from the community: How key population organizations in Vietnam transformed from peer support groups to clinical service providers | Pilot and scale up study of key population-led HIV services | Unassisted | Vietnam | Western Pacific | Lower Middle | Unclear | Non-RCT | Key populations | Peer educator |  |
| Wang et al. | 2020 | Correlates of HIV self-testing among female sex workers in China: implications for expanding HIV screening | Venue-based survey to identify associations with HIVST | Unassisted | China | Western Pacific | Upper Middle | Unclear | Non-RCT | FSW | Unclear | 2 weeks |
| Wang et al. | 2020 | Promoting oral HIV self‐testing via the internet among men who have sex with men in China: a feasibility assessment | Promotion of HIVST via the internet in the MSM population | Unassisted | China | Western Pacific | Upper Middle | Oral-fluid | Non-RCT | MSM | Facility | immediately |
| Wang et al. | 2018 | A randomized controlled trial evaluating efficacy of promoting a home-based HIV self-testing with online counseling on increasing HIV testing among men who have sex with men | Home-based HIV self-testing with pre-test/post-test counselling | Assisted | Hong kong | Western Pacific | High | Unclear | RCT | MSM | Online/mail |  |
| Wanga et al. | 2020 | Uptake and impact of facility-based HIV self-testing on PrEP delivery: a pilot study among young women in Kisumu, Kenya | Study of young women who were given the option to choose between provider-initiated testing and HIVST | Unassisted | Kenya | African | Lower Middle | Oral-fluid | RCT | Adolescents | Facility | same day |
| Wei et al. | 2018 | Which user errors matter during HIV self-testing? A qualitative participant observation study of men who have sex with men (MSM) in China | Unassisted HIVST conducted under the observation of a trained HIV testing counsellor | Unassisted | China | Western Pacific | Upper Middle | Both | Non-RCT | MSM | Facility |  |
| Widyanthini et al. | 2022 | HIV self-testing for men who have sex with men: an implementation trial in Indonesia | Supervised and unsupervised HIVST offered to MSM who did not have a HIV test result in the last 6 months | Unassisted | Indonesia | South-East Asian | Lower Middle | Oral-fluid | Non-RCT | Non-key population | Community |  |
| Wilson et al. | 2022 | High Acceptance and Completion of HIV Self-testing Among Diverse Populations of Young People in Kenya Using a Community-Based Distribution Strategy | Community-based HIVST services conducted by peer-mobilisers | Both | Kenya | African | Lower Middle | Oral-fluid | Non-RCT | Adolescents | Home-based testing, pharmacies, and ‘hotspots’ (bars/ nightclubs) |  |
| Witzel et al. | 2020 | HIV self‐testing intervention experiences and kit usability: results from a qualitative study among men who have sex with men in the SELPHI (Self‐Testing Public Health Intervention) randomized controlled trial in England and Wales | RCT of HIVST among MSM (SELPHI study) | Unassisted | UK | European | High | Fingerprick | Non-RCT | MSM | Facility | 24 hours |
| Wu et al. | 2021 | Social Media–Based Secondary Distribution of Human Immunodeficiency Virus/Syphilis Self-testing Among Chinese Men Who Have Sex with Men | Secondary distribution of HIVST kits by indexes through their networks | Unassisted | China | Western Pacific | Upper Middle | Fingerprick | Non-RCT | MSM | Online/mail |  |
| Wulandari et al. | 2020 | Uptake and acceptability of assisted and unassisted HIV self-testing among men who purchase sex in brothels in Indonesia: a pilot intervention study | Assisted or unassisted HIVST provided by lay health providers to men who purchase sex attending brothels | Both | Indonesia | South-East Asian | Lower Middle | Oral-fluid | RCT | Non-key population | Community | 2 months |
| Yang et al. | 2019 | HIV self-testing and potential linkage to care among men who have sex with men in China: A cross-sectional online survey | A nationwide online survey conducted to collect information on HIVST and source of kits | Unassisted | China | Western Pacific | Upper Middle | Unclear | Non-RCT | MSM | Unclear |  |
| Zhang et al. | 2020 | Impact of providing free HIV self-testing kits on frequency of testing among men who have sex with men and their sexual partners in China: A randomized controlled trial | MSM to distribute HIV self-testing kit to their sexual partners | Unassisted | China | Western Pacific | Upper Middle | Both | RCT | MSM | SNA |  |
| Zhang et al. | 2020 | HIV Self-Testing Programs to Men Who Have Sex with Men Delivered by Social Media Key Opinion Leaders and Community-Based Organizations are Both Effective and Complementary: A National Pragmatic Study in China | HIVST advertisements distributed by social media key opinion leaders and community-based organisations | Unassisted | China | Western Pacific | Upper Middle | Fingerprick | Non-RCT | MSM | Online/mail |  |
| Zhang et al. | 2021 | The longer-term effects of access to HIV self-tests on HIV testing frequency in high-risk gay and bisexual men: follow-up data from a randomised controlled trial | A waitlist RCT among MSM (FORTH trial) | Unassisted | Australia | Western Pacific | High | Oral-fluid | Non-RCT | MSM | Facility |  |
| Zhang, et al. | 2023 | The Impact of Providing Free HIV Self-testing on Sexual Risk Behaviors Among Men who have Sex with Men in Hunan, China: A Randomized Controlled Trial | RCT comparing facility-based testing vs facility- based HIV testing augmented with free HIVST | Unassisted | China | Western Pacific | Upper Middle | Fingerprick | RCT | MSM | Online/mail |  |
| Zhong et al. | 2017 | Acceptability and feasibility of a social entrepreneurship testing model to promote HIV self-testing and linkage to care among men who have sex with men | A social entrepreneurship testing model involving an online survey and paying a refundable deposit to receive an HIVST kit and a syphilis self-test kit | Unassisted | China | Western Pacific | Upper Middle | Fingerprick | Non-RCT | MSM | Online/mail |  |
| Zhou et al. | 2022 | Monetary incentives and peer referral in promoting secondary distribution of HIV self-testing among men who have sex with men in China: A randomized controlled trial | Secondary distribution program for HIVST involving monetary incentives and peer referral | Unassisted | China | Western Pacific | Upper Middle | Fingerprick | RCT | MSM | SNA |  |

AIDS, acquired immunodeficiency syndrome; ART, antiretroviral therapy; FSW, female sex workers; HIV, human immunodeficiency virus; HIVST, HIV self–testing; KP, key populations; MSM, men who have sex with men; NGO, nongovernmental organisation; PLWHIV, people living with HIV; PrEP, pre–exposure prophylaxis; PWID, people who inject drugs; RCT, randomised controlled trial; SNA, social network–based approach; STD, sexually transmitted diseases; STI, sexually transmitted infections

Figure S1. Forest plot for the proportion who were linked to confirmatory testing in unassisted HIVST

## Figure S2. Forest plot for proportion who were linked to confirmatory testing in assisted HIVST

Figure S3. Forest plot for proportion who were linked to ART initiation reports in unassisted HIVST

## Figure S4. Forest plot for proportion who were linked to ART initiation reports in assisted HIVST

## Figure S5. Forest plot for proportion who were linked to confirmatory testing from health facility delivery model

## Figure S6. Forest plot for proportion who were linked to confirmatory testing from community delivery model

## Figure S7. Forest plot for proportion who were linked to confirmatory testing from pharmacy delivery model

## **Figure S8.** Forest plot for proportion who were linked to confirmatory testing from online/mail delivery model

## Figure S9. Forest plot for proportion who were linked to confirmatory testing from peer-educator delivery model

## **Figure S10.** Forest plot for proportion who were linked to confirmatory testing from antenatal care delivery model

## Figure S11. Forest plot for proportion who were linked to confirmatory testing from other delivery models

## **Figure S12.** Forest plot for proportion who were linked to confirmatory testing with >1 type of delivery models

## **Figure S13.** Forest plot for proportion who were linked to ART initiation reports in RCT

## **Figure S14.** Forest plot for proportion who were linked to ART initiation reports in non-RCT

## Figure S15. Doi plot of included studies for linkage to confirmatory testing

## Figure S16. Doi plot of included studies for linkage to ART initiation

## Figure S17. Doi plot of included studies for linkage to care

## Figure S18. Doi plot of included studies of linkage to PrEP

## Table S3. Quality assessment of qualitative studies

| **Author** | **Year of Publication** | **Title** | **1. Is there congruity between the stated philosophical perspective and the research methodology? Yes/ No/ Unclear/ NA** | **2. Is there congruity between the research methodology and the research question or objectives? Yes/ No/ Unclear/ NA** | **3. Is there congruity between the research methodology and the methods used to collect data? Yes/ No/ Unclear/ NA** | **4. Is there congruity between the research methodology and the representation and analysis of data? Yes/ No/ Unclear/ NA** | **5. Is there congruity between the research methodology and the interpretation of results? Yes/ No/ Unclear/ NA** | **6. Is there a statement locating the researcher culturally or theoretically? Yes/ No/ Unclear/ NA** | **7. Is the influence of the researcher on the research, and vice- versa, addressed? Yes/ No/ Unclear/ NA** | **8. Are participants, and their voices, adequately represented? Yes/ No/ Unclear/ NA** | **9. Is the research ethical according to current criteria or, for recent studies, and is there evidence of ethical approval by an appropriate body? Yes/ No/ Unclear/ NA** | **10. Do the conclusions drawn in the research report flow from the analysis, or interpretation, of the data? Yes/ No/ Unclear/ NA** | **Overall appraisal: Include/ Exclude/ Seek further info** |
| --- | --- | --- | --- | --- | --- | --- | --- | --- | --- | --- | --- | --- | --- |
| Hubbard et al. | 2022 | Improving ART initiation among men who use HIV self-testing in Malawi: a qualitative study | Unclear | Unclear | Yes | Yes | Yes | No | No | Yes | Yes | Yes | Include |
| Koris et al. | 2021 | Youth-friendly HIV self-testing: Acceptability of campus-based oral HIV self-testing among young adult students in Zimbabwe | Unclear | Unclear | Yes | Yes | Yes | No | No | Yes | Yes | Yes | Include |
| Kumwenda et al. | 2019 | Post-test adverse psychological effects and coping mechanisms amongst HIV self-tested individuals living in couples in urban Blantyre, Malawi | Yes | Yes | Yes | Yes | Yes | No | No | Yes | Yes | Yes | Include |
| Robson et al. | 2020 | A community-based intervention (men's spaces) to engage men in HIV and sexual health services in Malawi: a pilot study | Unclear | Unclear | Yes | Yes | Yes | No | No | Unclear | Yes | Yes | Include |
| Witzel et al. | 2020 | HIV self‐testing intervention experiences and kit usability: results from a qualitative study among men who have sex with men in the SELPHI (Self‐Testing Public Health Intervention) randomized controlled trial in England and Wales | Unclear | Unclear | Yes | Yes | Yes | No | No | Yes | Yes | Yes | Include |

ART, antiretroviral therapy; HIV, human immunodeficiency virus

## Table S4. Quality assessment of randomised controlled trial studies

| **Author** | **Year of Publication** | **Title** | **1. Risk from Randomisation Process?** | **2. deviations from the intended interventions?** | **3. Missing Outcome data?** | **4. Bias Outcome measurement?** | **5. Bias in selection of reported result?** | **Overall Risk** | **Comments** |
| --- | --- | --- | --- | --- | --- | --- | --- | --- | --- |
| Carballo-Diéguez et al. | 2020 | Use of Rapid HIV Self-Test to Screen Potential Sexual Partners: Results of the ISUM Study | Low | Some concerns | Low | Low | Low | Some concerns | self-reported HIV testing for primary outcome |
| Chanda et al. | 2017 | HIV self-testing among female sex workers in Zambia: a randomized controlled trial | Low | Some concerns | Low | Some concerns | Low | High | proxy reporting of the male partner testing outcome |
| Choko et al. | 2019 | HIV self-testing alone or with additional interventions, including financial incentives, and linkage to care or prevention among male partners of antenatal care clinic attendees in Malawi: An adaptive multi-arm, multi-stage cluster randomised trial | Low | Some concerns | Low | Low | Low | Some concerns | reported data of male testing by interview the female partner |
| Choko et al. | 2021 | Partner-delivered HIV self-test kits with and without financial incentives in antenatal care and index patients with HIV in Malawi: a three-arm, cluster-randomised controlled trial | Low | Some concerns | Low | Some concerns | Low | High | abstract |
| Choko et al. | 2015 | Uptake, Accuracy, Safety, and Linkage into Care over Two Years of Promoting Annual Self-Testing for HIV in Blantyre, Malawi: A Community-Based Prospective Study | Low | Some concerns | Low | Some concerns | Low | High | systematic sample of HIVST participants selected for home visit by study nurses for quality assurance |
| De Schacht et al. | 2022 | Reaching Men and Young Adults in a Pharmacy-Based HIV Self-Testing Strategy: Results from an Acceptability Study in Mozambique | Low | Some concerns | Some concerns | Some concerns | Low | High | some of the study staff gave some control participants an HIVST kit based on perceived need |
| Dovel et al. | 2020 | Effect of facility-based HIV self-testing on uptake of testing among outpatients in Malawi: a cluster-randomised trial | Low | Some concerns | Low | Some concerns | Low | High | high level of attrition for survey contact; self-reporting data on test confirmation |
| Dovel, et al. | 2023 | Effect of index HIV self-testing for sexual partners of clients enrolled in antiretroviral therapy (ART) programs in Malawi: A randomized controlled trial | Low | Some concerns | Some concerns | Some concerns | Low | High | rely on secondary reports from ART clients to determine primary outcomes for their sexual partners |
| Floyd et al. | 2018 | Linkage to HIV care following HIV self-testing: a cluster randomised trial of community-based distribution of oral HIV self-test kits nested in four HPTN 071 communities in Zambia | Low | Some concerns | Low | Some concerns | Low | High | self-reported HIV testing and result data; unable to mask the interventions form research assistants who did the exit surveys |
| Indravudh et al. | 2021 | Effect of community-led delivery of HIV self-testing on HIV testing and antiretroviral therapy initiation in Malawi: A cluster-randomised trial | Low | Some concerns | Low | Some concerns | Low | High |  |
| Jamil et al. | 2017 | Effect of availability of HIV self-testing on HIV testing frequency in gay and bisexual men at high risk of infection (FORTH): a waiting-list randomised controlled trial | Low | Some concerns | Low | Some concerns | Low | High | abstract |
| Joseph Davey et al. | 2022 | HIV testing and linkage to ART following secondary distribution of HIV self-test kits to male partners of women living with HIV: a pilot randomized control trial in Mpumalanga, South Africa | Low | Some concerns | Low | Some concerns | Low | High | self-reported HIV testing outcomes; lack of baseline measurement for the primary outcome |
| Katz et al. | 2018 | HIV self-testing increases HIV testing frequency in high-risk men who have sex with men: a randomized controlled trial | Low | Some concerns | Some concerns | Low | Low | High | self-reported testing data |
| Kimaru et al. | 2018 | HIV self-test distribution by young women: an innovative way to reach men in rural South Africa | Low | Some concerns | Some concerns | Some concerns | Low | High | some of the study staff gave some Control participants an HIVST kit based on perceived need |
| Konda et al. | 2022 | HIV self-testing to increase combination prevention demand among men who have sex with men (MSM) and transgender women (TGW): a randomized clinical trial and sub-study of the ImPrEP project | Low | Some concerns | Low | Some concerns | Low | High | high level of attrition in 9-month survey; self-tests reported by participants were validated by medical records |
| Korte et al. | 2020 | HIV oral self-testing for male partners of women attending antenatal care in central Uganda: uptake of testing and linkage to care in a randomized trial | Low | Some concerns | Low | Some concerns | Low | High | abstract |
| Lippman et al. | 2018 | High Acceptability and Increased HIV-Testing Frequency After Introduction of HIV Self-Testing and Network Distribution Among South African MSM | Low | Some concerns | Low | Some concerns | Low | High | abstract |
| MacGowan et al. | 2020 | Effect of internet-distributed HIV self-tests on HIV diagnosis and behavioral outcomes in men who have sex with men: a randomized clinical trial | Low | Some concerns | Some concerns | Some concerns | Low | High | self-reported partner HIV testing data |
| MacPherson et al. | 2014 | Effect of optional home initiation of HIV care following HIV self-testing on antiretroviral therapy initiation among adults in Malawi: a randomized clinical trial | Low | Some concerns | Low | Some concerns | Low | High | all measures based on self-report during surveys and received HIVST logs |
| Majam, et al. | 2022 | Incentives to promote accessing HIV care and viral suppression among HIV self-screening test users who obtain a reactive result | Low | Some concerns | Some concerns | Some concerns | Low | High | high attrition rates in follow-ups; self-reported outcome data |
| Masters et al. | 2016 | Promoting partner testing and couples testing through secondary distribution of HIV self-tests: a randomized clinical trial | Low | Some concerns | Low | Some concerns | Low | High | abstract |
| Mavhu et al. | 2021 | Innovative demand creation strategies to increase voluntary medical male circumcision uptake: a pragmatic randomised controlled trial in Zimbabwe | Low | Some concerns | Some concerns | Some concerns | Low | High | reporting results was optional and not required |
| Mulubwa et al. | 2019 | Community based distribution of oral HIV self-testing kits in Zambia: a cluster-randomised trial nested in four HPTN 071 (PopART) intervention communities | Low | Some concerns | Low | Some concerns | Low | High |  |
| Mutale et al. | 2021 | Addition of HIV self-test kits to partner notification services to increase HIV testing of male partners of pregnant women in Zambia: two parallel randomised trials | Low | Some concerns | Low | Some concerns | Low | High | some high attrition levels; additional data on HIVST use obtained from a phone-administered follow-up questionnaire with clients |
| Neuman et al. | 2021 | Does community-based distribution of HIV self-tests increase uptake of HIV testing? Results of pair-matched cluster randomised trial in Zambia | Low | Some concerns | Low | Some concerns | Low | High |  |
| Oldenburg et al. | 2018 | Brief Report: intimate partner violence and antiretroviral therapy initiation among female sex workers newly diagnosed with HIV in Zambia: a prospective study | Low | Some concerns | Low | Some concerns | Low | High | data reported by female participants about their male partner HIV testing outcomes |
| Ortblad et al. | 2017 | Direct provision versus facility collection of HIV self-tests among female sex workers in Uganda: A cluster-randomized controlled health systems trial | Low | Some concerns | Low | Some concerns | Low | High | HIV testing outcomes measured using self-reported data |
| Pai et al. | 2021 | Impact of a personalised, digital, HIV self-testing app-based program on linkages and new infections in the township populations of South Africa | Some concerns | Some concerns | Low | Low | Low | High | all measures were self-reported and may be subjected to social desirability or other bias |
| Rodger | 2022 | Free HIV self-test for identification and linkage to care of previously undetected HIV infection in men who have sex with men in England and Wales (SELPHI): an open-label, internet-based, randomised controlled trial | Low | Some concerns | Low | Low | Low | Some concerns | participants did not interpret self-test image results in a random order |
| Sibanda et al. | 2021 | Comparison of community-led distribution of HIV self-tests kits with distribution by paid distributors: a cluster randomised trial in rural Zimbabwean communities | Low | Some concerns | Low | Some concerns | Low | High | self-reported outcomes |
| Sibanda et al. | 2021 | Community-based HIV self-testing: a cluster-randomised trial of supply-side financial incentives and time-trend analysis of linkage to antiretroviral therapy in Zimbabwe | Low | Some concerns | Low | Some concerns | Low | High | quasi-randomised trial; data from the app were corroborated with lab data and clinic data |
| Stephenson et al. | 2022 | Efficacy of a telehealth delivered couples' HIV counseling and testing (chtc) intervention to improve formation and adherence to safer sexual agreements among male couples in the us: results from a randomized control trial | Some concerns | Some concerns | High | Some concerns | Low | High | the use of self-reports for primary outcomes, but minimised the bias by use of ACASI |
| Stephenson et al. | 2020 | Project Moxie: Results of a feasibility study of a telehealth intervention to increase HIV testing among binary and nonbinary transgender youth | Low | Some concerns | Low | Some concerns | Low | High | potential bias from trial outcomes based on self- report, but used ACASI to minimise this bias risk |
| Tonen-Wolyec et al. | 2020 | Comparison of practicability and effectiveness between unassisted HIV self-testing and directly assisted HIV self-testing in the Democratic Republic of the Congo: a randomized feasibility trial | Low | Some concerns | Low | Some concerns | Low | High | participants in the intervention arm over-reported data on the safety of their sexual agreements is possible |
| Wang et al. | 2018 | A randomized controlled trial evaluating efficacy of promoting a home-based HIV self-testing with online counseling on increasing HIV testing among men who have sex with men | Low | Some concerns | Low | Some concerns | Low | High | two significant differences in the baseline identified in post-randomisation; sub-optimal retention rates, particularly lower in the intervention arm; self-reported follow-up surveys |
| Wulandari et al. | 2020 | Uptake and acceptability of assisted and unassisted HIV self-testing among men who purchase sex in brothels in Indonesia: a pilot intervention study | Low | Some concerns | Low | Some concerns | Low | High | abstract |
| Zhang et al. | 2020 | Impact of providing free HIV self-testing kits on frequency of testing among men who have sex with men and their sexual partners in China: A randomized controlled trial | Low | Some concerns | Low | Some concerns | Low | High | self-reported outcomes |
| Zhang, et al. | 2023 | The impact of providing free HIV self-testing on sexual risk behaviors among men who have sex with men in Hunan, China: a randomized controlled trial | Low | Some concerns | Low | Some concerns | Low | High | delayed re-reading of used oral self-tests by the research team is not currently a valid methodological approach to ensure quality and monitoring which may overestimate true HIV positivity |
| Zhou et al. | 2022 | Monetary incentives and peer referral in promoting secondary distribution of HIV self-testing among men who have sex with men in China: A randomized controlled trial | Low | Some concerns | Low | Some concerns | Low | High | data on SBHT numbers were based on self-report by the participants, but self-reported number of HIVST in the intervention arm was validated; the number of partner HIV testing was indirectly reported by participants |
| Zhu et al. | 2019 | Effects of a mobile health intervention to promote HIV self-testing with MSM in China: a randomized controlled trial | Low | Some concerns | Low | Low | Low | Some concerns |  |

AIDS, acquired immunodeficiency syndrome; ART, antiretroviral therapy; FSW, female sex workers; HIV, human immunodeficiency virus; HIVST, HIV self–testing; KP, key populations; MSM, men who have sex with men; NGO, nongovernmental organisation; PLWHIV, people living with HIV; PrEP, pre–exposure prophylaxis; PWID, people who inject drugs; RCT, randomised controlled trial; SNA, social network–based approach; STD, sexually transmitted diseases

## Table S5. Quality assessment of quasi-experimental studies

| **Author** | **Year of Publication** | **Title** | **1. Is it clear in the study what is the ‘cause’ and what is the ‘effect’ (i.e. there is no confusion about which variable comes first)? Yes/ No/ Unclear/ NA** | **2. Were the participants included in any comparisons similar? Yes/ No/ Unclear/ NA** | **3. Were the participants included in any comparisons receiving similar treatment/care, other than the exposure or intervention of interest? Yes/ No/ Unclear/ NA** | **4. Was there a control group? Yes/ No/ Unclear/ NA** | **5. Were there multiple measurements of the outcome both pre and post the intervention/exposure? Yes/ No/ Unclear/ NA** | **6. Was follow up complete and if not, were differences between groups in terms of their follow up adequately described and analyzed? Yes/ No/ Unclear/ NA** | **7. Were the outcomes of participants included in any comparisons measured in the same way? Yes/ No/ Unclear/ NA** | **8. Were outcomes measured in a reliable way? Yes/ No/ Unclear/ NA** | **9. Was appropriate statistical analysis used? Yes/ No/ Unclear/ NA** | **Overall appraisal: Include/ Exclude/ Seek further info** |
| --- | --- | --- | --- | --- | --- | --- | --- | --- | --- | --- | --- | --- |
| Choko et al. | 2018 | A pilot trial of the peer-based distribution of HIV self-test kits among fishermen in Bulisa, Uganda | Yes | NA | NA | No | No | Yes | Yes | Yes | Yes | Include |
| Kitenge et al. | 2020 | Unassisted peer-based distribution of HIV oral self-testing for the hard to reach in rural KwaZulu natal, South Africa | Yes | NA | NA | No | Yes | Yes | Yes | Yes | Yes | Include |
| Tonen-Wolyec et al. | 2021 | Uptake of HIV/AIDS Services following a positive self-test is lower in men than women in the Democratic Republic of the Congo | Yes | NA | NA | No | No | Yes | Yes | Yes | Yes | Include |
| Wanga et al. | 2020 | Uptake and impact of facility-based HIV self-testing on PrEP delivery: a pilot study among young women in Kisumu, Kenya | Yes | NA | NA | No | Yes | Yes | Yes | Yes | Yes | Include |
| Wulandari et al. | 2020 | Uptake and acceptability of assisted and unassisted HIV self-testing among men who purchase sex in brothels in Indonesia: a pilot intervention study | Yes | NA | NA | No | Yes | Yes | Yes | Yes | Yes | Include |

AIDS, acquired immunodeficiency syndrome; ART, antiretroviral therapy; FSW, female sex workers; HIV, human immunodeficiency virus; HIVST, HIV self–testing; KP, key populations; MSM, men who have sex with men; NGO, nongovernmental organisation; PLWHIV, people living with HIV; PrEP, pre–exposure prophylaxis; PWID, people who inject drugs; RCT, randomised controlled trial; SNA, social network–based approach; STD, sexually transmitted diseases

## Table S6. Quality assessment for cross-sectional studies

| **Author** | **Year of Publication** | **Title** | **1. Were the criteria for inclusion in the sample clearly defined? Yes/ No/ Unclear/ NA** | **2. Were the study subjects and the setting described in detail? Yes/ No/ Unclear/ NA** | **3. Was the exposure measured in a valid and reliable way? Yes/ No/ Unclear/ NA** | **4. Were objective, standard criteria used for measurement of the condition? Yes/ No/ Unclear/ NA** | **5. Were confounding factors identified? Yes/ No/ Unclear/ NA** | **6. Were strategies to deal with confounding factors stated? Yes/ No/ Unclear/ NA** | **7. Were the outcomes measured in a valid and reliable way? Yes/ No/ Unclear/ NA** | **8. Was appropriate statistical analysis used? Yes/ No/ Unclear/ NA** | **Overall appraisal: Include/ Exclude/ Seek further info** |
| --- | --- | --- | --- | --- | --- | --- | --- | --- | --- | --- | --- |
| Adepoju et al. | 2022 | How efficient are HIV self-testing models? A comparison of community, facility, one-stop-shop and pharmacy retail distribution models in Nigeria | No | No | Unclear | Unclear | Unclear | N/A | Unclear | Yes | Exclude |
| Altaf et al. | 2022 | Acceptability and feasibility of HIV self-testing among transgender people in Larkana, Pakistan: Results from a pilot project | Yes | Yes | Yes | Yes | Yes | No | Yes | Yes | Include |
| Altaf, et al. | 2023 | Acceptability and feasibility of intra-peer and social media approach for HIV self-testing among men who have sex with men in Karachi: lessons from a pilot study | Unclear | Yes | Yes | Yes | No | No | Yes | Yes | Include |
| Anand et al. | 2017 | Implementation of an online HIV prevention and treatment cascade in Thai men who have sex with men and transgender women using Adam's Love Electronic Health Record system | Yes | Yes | Yes | Yes | Yes | Yes | Yes | Yes | Include |
| Bbuye et al. | 2022 | Factors associated with linkage to HIV care among oral self-tested HIV positive adults in Uganda | Yes | Yes | Yes | Yes | No | No | Yes | Yes | Include |
| Bell et al. | 2021 | Online HIV self-testing (HIVST) dissemination by an australian community peer HIV organisation: A scalable way to increase access to testing, particularly for suboptimal testers | Yes | Yes | Yes | Yes | Yes | No | Yes | Yes | Include |
| Belza et al. | 2012 | Supervised blood-based self-sample collection and rapid test performance: a valuable alternative to the use of saliva by HIV testing programmes with no medical or nursing staff | No | No | Unclear | Unclear | No | No | Unclear | Yes | Exclude |
| Bil et al. | 2019 | Usage of purchased self-Tests for HIV infections among migrants living in the UK, France and the Netherlands: A cross-sectional study | Yes | Yes | Unclear | Unclear | Yes | No | No | Yes | Include |
| Boni et al. | 2019 | An internet-based HIV self-testing program to increase HIV testing uptake among men who have sex with men in Brazil: descriptive cross-sectional analysis | Yes | Yes | Yes | Yes | Yes | No | Unclear | Yes | Include |
| Brady et al. | 2017 | HIV self-testing: Feasibility and acceptability of a large scale national service | No | No | Unclear | Unclear | Unclear | Unclear | Unclear | Yes | Exclude |
| Cary et al. | 2017 | HIV self-testing: feasibility and acceptability of a large-scale national service delivered by a community organization | Unclear | Unclear | Yes | No | Unclear | Unclear | No | NA | Exclude |
| Cassell et al. | 2022 | A cross-sectional assessment of HIV self-testing preferences and uptake among key populations in Phnom Penh, Cambodia | Yes | Yes | No | Yes | No | No | Yes | Unclear | Include |
| Chan et al. | 2021 | Effectiveness of a novel HIV self-testing service with online real-time counseling support (HIVST-online) in increasing HIV testing rate and repeated HIV testing among men who have sex with men in Hong Kong: Results of a pilot implementation project | Yes | Yes | Yes | Yes | Yes | No | Yes | Yes | Include |
| Chesang et al. | 2016 | Using peer educators to scale-up HIV oral self-testing among female sex workers: An implementation science approach from Kenya | No | No | No | Yes | No | No | Yes | Unclear | Include |
| Conserve, et al. | 2023 | Peer-based Promotion and Nurse-led Distribution of HIV Self-Testing Among Networks of Men in Dar es Salaam, Tanzania: Development and Feasibility Results of the STEP Intervention | Yes | Yes | Yes | Yes | No | Yes | Yes | Yes | Include |
| da Cruz et al. | 2021 | Comprehensive approach to HIV/AIDS testing and linkage to treatment among men who have sex with men in Curitiba, Brazil | Unclear | Yes | Unclear | Unclear | No | N/A | Unclear | Yes | Include |
| De Boni et al. | 2018 | Self-testing, communication and information technology to promote HIV diagnosis among young gay and other men who have sex with men (MSM) in Brazil | Yes | Yes | Yes | No | No | No | Unclear | Yes | Include |
| Denisiuk, et al. | 2023 | Social Network Strategy improves access to HIV testing and harm reduction programs for PWID and their partners in Kazakhstan | Yes | Yes | Yes | Yes | No | Yes | Yes | Yes | Include |
| DiCarlo et al. | 2020 | HIV self-testing reaches previously unreached key and vulnerable populations in Democratic Republic of the Congo (DRC) | Yes | Yes | Yes | Yes | Unclear | Unclear | Yes | Unclear | Exclude |
| Edelstein et al. | 2020 | Five waves of an online hiv self-test giveaway in New York City, 2015 to 2018 | Yes | Yes | Yes | Yes | No | No | Yes | Yes | Include |
| Eustaquio et al. | 2022 | Outcomes of a community-led online-based HIV self-testing demonstration among cisgender men who have sex with men and transgender women in the Philippines during the COVID-19 pandemic: a retrospective cohort study | Yes | Yes | Yes | Yes | Yes | Yes | Yes | Yes | Include |
| Fernandez et al. | 2022 | High linkage to treatment among key populations who self-test through a peer HIV self-test distribution and community-based ART program among key populations in Lagos, Nigeria | Yes | Yes | Yes | Yes | Unclear | Unclear | Yes | Unclear | Exclude |
| Gashobotse et al. | 2019 | Peer-mobilized HIV self-testing increases case detection and linkage to ART among key populations in Burundi | Unclear | Unclear | Yes | Yes | Unclear | Unclear | Yes | Yes | Exclude |
| Girault et al. | 2021 | Uptake of oral fluid-based HIV self-testing among men who have sex with men and transgender women in Thailand | Yes | Yes | Yes | Yes | Yes | No | Yes | Yes | Include |
| Greacen et al. | 2012 | Access to and use of unauthorised online HIV self-tests by internet-using French-speaking men who have sex with men | Yes | Yes | Yes | Unclear | Yes | Unclear | Unclear | Yes | Exclude |
| Green et al. | 2018 | From conventional to disruptive: upturning the HIV testing status quo among men who have sex with men in Vietnam | Yes | Yes | Yes | Yes | Yes | Yes | Yes | Yes | Include |
| Guadamuz et al. | 2022 | A pilot feasibility study of HIV self-testing with online supervision among MSM who attend sexualized drug parties in Thailand | Yes | Yes | Yes | Yes | Unclear | Unclear | Yes | Unclear | Exclude |
| Hacking et al. | 2022 | HIV Self-testing Among Previously Diagnosed HIV-Positive People in Khayelitsha, South Africa: No Evidence of Harm but may Facilitate Re-engagement in ART Care | Yes | Yes | No | Yes | No | No | Yes | Unclear | Include |
| Hector et al. | 2018 | Acceptability and performance of a directly assisted oral HIV self-testing intervention in adolescents in rural Mozambique | Yes | Yes | No | Yes | No | No | Yes | Unclear | Include |
| Horvath et al. | 2021 | Histep: A single-arm pilot study of a technology-assisted HIV self-testing intervention in Kampala, Uganda | Yes | Yes | Yes | Yes | No | No | Yes | Yes | Include |
| Huang et al. | 2016 | Using Grindr, a smartphone social-networking application, to increase HIV self-testing among black and Latino men who have sex with men in Los Angeles, 2014 | Yes | Yes | Yes | Unclear | No | No | No | Yes | Include |
| Huang et al. | 2022 | Towards the first 90: impact of the national HIV self-test program on case finding and factors associated with linkage to confirmatory diagnosis in Taiwan | Yes | Yes | Yes | Yes | Unclear | Unclear | Yes | Yes | Include |
| Jin et al. | 2019 | An internet-based self-testing model (easy test): cross-sectional survey targeting men who have sex with men who never tested for HIV in 14 provinces of China | Yes | Yes | No | Yes | No | No | Yes | Unclear | Include |
| Johnson et al. | 2022 | Combating stigma through HIV self-testing: New York state's HIV home test giveaway program for sexual minorities | Yes | Yes | No | Yes | No | No | Yes | NA | Include |
| Junior Matovu et al. | 2022 | Acceptability, willingness to use and preferred distribution models of oral-based HIV self-testing kits among key and priority populations enrolled in HIV pre-exposure prophylaxis clinics in central Uganda. A mixed-methods cross-sectional study | No | Yes | Yes | Yes | No | No | Yes | Yes |  |
| Khajehkazemi et al. | 2021 | Feasibility of HIV self-testing among female sex workers in Iran: the SELFii study | Yes | No | No | Yes | No | No | Yes | Unclear | Include |
| Khanal et al. | 2020 | From a demonstration study to national rollout: HIV self-testing in nepal | No | No | Unclear | No | No | No | Unclear | Unclear | Exclude |
| Kikuli, et al. | 2023 | Peer client bond in accelerating the return of unassisted HIV self-test kits among female sex workers. Afya jumuishi Geita region | No | No | Unclear | Unclear | Unclear | Unclear | Unclear | Unclear | Exclude |
| Kitenge et al. | 2022 | Acceptability of unsupervised peer-based distribution of HIV oral self-testing for the hard-to-reach in rural KwaZulu Natal, South Africa: Results from a demonstration study | Yes | Yes | Yes | Yes | No | N/A | Yes | Yes | Include |
| Kra, et al. | 2023 | HIV self-testing positivity rate and linkage to confirmatory testing and care: a telephone survey in Cote d'Ivoire, Mali and Senegal | Yes | Yes | Yes | Yes | No | No | Yes | Yes | Include |
| Kwan, et al. | 2023 | Implementation cascade of a social network-based HIV self-testing approach for men who have sex with men: cross-sectional study | Yes | Yes | Yes | Yes | No | No | Yes | Yes | Include |
| Ky-Zerbo, et al. | 2022 | I take it and give it to my partners who will give it to their partners: Secondary distribution of HIV self-tests by key populations in Cote d'Ivoire, Mali, and Senegal | Yes | Yes | Yes | Yes | No | No | Yes | Yes | Include |
| Larsson, et al. | 2023 | Feasibility and usability of mobile technology to assist HIV self-testing in youth in Zimbabwe: a mixed-methods study | Unclear | Yes | Yes | Yes | No | No | Yes | Yes | Include |
| Lavoie et al. | 2022 | Scale-up and optimization of HIV self-testing for the last mile in Botswana | Unclear | Yes | Yes | Yes | Unclear | Unclear | Yes | Unclear | Exclude |
| Lebina et al. | 2019 | Feasibility of community-based HIV self-screening in South Africa: a demonstration project | Yes | Yes | Yes | Yes | No | No | Yes | Yes | Include |
| Leontieva et al. | 2022 | Advancing peer-driven social network testing (SNT) and index testing to maximize reach of HIV testing services (HTS) among men who have sex with men (MSM) and their contacts in Ukraine | Yes | Yes | Yes | Yes | Unclear | Unclear | Yes | Unclear | Exclude |
| Leontieva et al. | 2022 | Pragmatically approaching social network testing (SNT): using a peer-driven community outreach model to extend reach of HIV testing services (HTS) to networks of people who inject drugs (PWID) in Ukraine | Unclear | Unclear | Yes | Yes | Unclear | Unclear | Yes | Unclear | Exclude |
| Li et al. | 2021 | Feasibility of indirect secondary distribution of HIV self-test kits via WeChat among men who have sex with men: national cross-sectional study in China | Yes | Yes | Yes | Yes | Yes | Yes | Yes | Yes | Include |
| Lillie et al. | 2021 | Increasing testing options for key populations in Burundi through peer-assisted HIV self-testing: descriptive analysis of routine programmatic data | Yes | Yes | No | Yes | No | No | Yes | Yes | Include |
| Lyons et al. | 2019 | Use and acceptability of HIV self-testing among first-time testers at risk for HIV in Senegal | Yes | Yes | Yes | Yes | No | N/A | Yes | Yes | Include |
| Lyu, et al. | 2023 | Factors associated with first-time HIV testing among MSM via secondary distribution of HIV self-test kits in Zhuhai, China | Yes | Yes | Yes | Yes | Yes | Unclear | Yes | Yes | Include |
| Maatouk et al. | 2021 | Community-led HIV self-testing for men who have sex with men in Lebanon: lessons learned and impact of COVID-19 | Unclear | No | Unclear | Unclear | Unclear | No | No | Yes | Include |
| Majam et al. | 2019 | High self-reporting of HIV self-test results through an interactive voice response telephone line in inner city Johannesburg | Unclear | Unclear | Yes | No | Unclear | Unclear | No | Unclear | Exclude |
| Marlin et al. | 2014 | Piloting an HIV self-test kit voucher program to raise serostatus awareness of high-risk African Americans, Los Angeles | Yes | Yes | Yes | Yes | No | N/A | Yes | Yes | Include |
| Matovu et al. | 2020 | Feasibility and acceptability of a pilot, peer-led HIV self-testing intervention in a hyperendemic fishing community in rural Uganda | Yes | Yes | Yes | Yes | Yes | No | Yes | Yes | Include |
| Matsuda, et al. | 2022 | Impact of covid-19 on people living with HIV-1: care and prevention indicators at a local and nationwide level, Santo Andre, Brazil | Unclear | Yes | Yes | Yes | No | No | Yes | Yes | Include |
| Mavedzenge et al. | 2017 | Feasibility of HIV self-test programming among female sex workers in Zimbabwe | Unclear | Yes | Yes | Yes | Unclear | Unclear | Unclear | Unclear | Exclude |
| McHugh, et al. | 2023 | On campus HIV self-testing distribution at tertiary level colleges in Zimbabwe increases access to HIV testing for youth | Yes | Yes | Yes | Yes | Yes | Unclear | Yes | Yes | Include |
| Medley et al. | 2019 | Outcome and cost of 3 methods for increasing male partner testing in South Africa | Yes | Yes | No | Yes | No | No | Yes | Unclear | Include |
| Mee et al. | 2021 | Factors associated with the successful distribution of HIV self-tests through routine clinical settings in Lusaka, Zambia: a cohort study | Unclear | Yes | Yes | Yes | Unclear | Unclear | No | Yes | Exclude |
| Mkopi, et al. | 2023 | Acceptability and uptake of oral HIV self-testing among rural community members in Tanzania: a pilot study | Yes | Yes | Yes | Yes | Yes | No | Yes | Yes | Include |
| Moore et al. | 2019 | Investigating the addition of oral HIV self-tests among populations with high testing coverage - Do they add value? Lessons from a study in Khayelitsha, South Africa | Yes | Yes | Yes | Yes | No | No | Yes | Yes | Include |
| Moore et al. | 2017 | Self-testing: an effective means of increasing HIV-testing and status awareness | Unclear | Unclear | Yes | Yes | Unclear | Unclear | Yes | Unclear | Exclude |
| Mshweshwe-Pakela et al. | 2022 | Digitally supported HIV self-testing increases facility-based HIV testing capacity in Ekurhuleni, South Africa | Yes | Yes | Yes | Yes | Yes | No | Yes | Yes | Include |
| Muchedzi et al. | 2021 | High HIV positivity rates following large-scale HIV self-testing implementation in Zimbabwe, 2018-2020 | Yes | Yes | Yes | Yes | No | No | Yes | Yes | Include |
| Mugo et al. | 2017 | Uptake and acceptability of oral HIV self-testing among community pharmacy clients in Kenya: A feasibility study | Yes | Yes | Yes | Yes | Yes | No | Yes | Yes | Include |
| Mwanda et al. | 2021 | Extending reach of HIV testing services (HTS) through private-sector outlets: feasibility of offering HIV self-testing (HIVST) at pharmacies and alternative medicine centers (AMC) in Democratic Republic of the Congo (DRC) | Unclear | Yes | Yes | Yes | Unclear | Unclear | Unclear | Unclear | Exclude |
| Nangendo, et al. | 2023 | Village health team-delivered oral HIV self-testing increases linkage-to-care and antiretroviral-therapy initiation among men in Uganda | Yes | Yes | Yes | Yes | Yes | No | Yes | Yes | Include |
| Napierala et al. | 2019 | HIV self-testing services for female sex workers, Malawi and Zimbabwe | No | No | Yes | Yes | No | No | Yes | Unclear |  |
| Nasuuna et al. | 2022 | Early observations from the HIV self-testing program among key populations and sexual partners of pregnant mothers in Kampala, Uganda: a cross sectional study | Yes | Yes | No | Yes | No | No | Yes | Unclear | Include |
| Nguyen et al. | 2019 | Community-led HIV testing services including HIV self-testing and assisted partner notification services in Vietnam: lessons from a pilot study in a concentrated epidemic setting | Yes | Yes | No | Yes | No | No | Yes | Unclear | Include |
| Nguyen et al. | 2022 | HIV self-testing reached our key populations regardless of COVID-19 | Unclear | Yes | Yes | Yes | Unclear | Unclear | Yes | Unclear | Exclude |
| Ngwalle, et al. | 2023 | Successful return of client's results after an HIV self-test: The role of peer educators in southern highlands, Tanzania | No | No | Unclear | Unclear | Unclear | Unclear | Unclear | Unclear | Exclude |
| O'Byrne et al. | 2021 | At-home HIV self-testing during COVID: implementing the GetaKit project in Ottawa | No | Yes | No | Yes | No | No | Yes | Unclear | Include |
| Okoboi et al. | 2020 | Peer distribution of HIV self-test kits to men who have sex with men to identify undiagnosed HIV infection in Uganda: A pilot study | Yes | Yes | No | Yes | No | No | Yes | Yes | Include |
| Owusu et al. | 2020 | Acceptability of provider assisted self-testing among men who have sex with men in chana | Yes | Yes | Yes | Yes | Unclear | Unclear | No | Unclear | Exclude |
| Pai et al. | 2013 | Will an unsupervised self-testing strategy for HIV work in health care workers of South Africa? A cross sectional pilot feasibility study | Yes | Yes | Yes | Yes | No | No | Yes | Yes | Include |
| Pai et al. | 2018 | An unsupervised smart app-optimized HIV self-testing program in Montreal, Canada: cross-sectional study | Yes | Yes | Yes | Yes | No | No | Yes | Yes | Include |
| Pasipamire et al. | 2020 | Implementation of community and facility-based HIV self-testing under routine conditions in southern Eswatini | Yes | Yes | Yes | Yes | Yes | No | Yes | Yes | Include |
| Phanuphak et al. | 2020 | Linkages to HIV confirmatory testing and antiretroviral therapy after online, supervised, HIV self-testing among Thai men who have sex with men and transgender women | Yes | Yes | Yes | Yes | Yes | Yes | Yes | Yes | Include |
| Phatsoane Gaven, et al. | 2023 | HIV self-test reporting using mHealth platforms: A pilot study in Johannesburg, South Africa | Yes | Yes | Yes | Yes | Yes | No | Yes | Yes | Include |
| Phongphiew et al. | 2021 | Acceptability of blood-based HIV self-testing among adolescents aged 15–19 years at risk of HIV acquisition in Bangkok | Yes | Yes | Yes | Yes | No | No | Yes | Yes | Include |
| Pisemskiy | 2018 | Effective promotion of HIV Self-testing among MSM in Russia in the context of growing stigma and discrimination | No | No | Unclear | Unclear | No | No | Unclear | Unclear | Exclude |
| Pollard et al. | 2022 | Tailoring HIVST to local communities can improve uptake: lessons learned from a virtual HIV self-testing intervention in India | Unclear | Yes | Yes | Yes | Unclear | Unclear | Yes | Unclear | Exclude |
| Qin et al. | 2016 | Benefits and adverse outcomes of HIV self-testing among high-risk MSM in China: an implementation perspective | Yes | Yes | Yes | Yes | Yes | Yes | Yes | Yes | Include |
| Ren et al. | 2017 | HIV care-seeking behaviour after HIV self-testing among men who have sex with men in Beijing, China: A cross-sectional study | Yes | Yes | Yes | Yes | No | No | Yes | Yes | Include |
| Rosadiño et al. | 2022 | SelfCare's Cat and Pao: the use of community-led automated virtual assistance and peer support in an unassisted HIV self-testing program in Metro Manila, Philippines | Yes | Yes | Yes | Yes | Unclear | Unclear | Yes | Unclear | Exclude |
| Rosengren et al. | 2016 | Feasibility of using GrindrTM to distribute HIV self-test kits to men who have sex with men in Los Angeles, California | Yes | Yes | Yes | Yes | No | No | Yes | Yes | Include |
| Rosenthal, et al. | 2023 | Geospatial prioritization to reach Hispanic or Latino and other priority populations through HIV home testing services | Unclear | Yes | Yes | Yes | No | No | Yes | Yes | Include |
| Salvadori, et al. | 2022 | Uptake, acceptability and interpretability of 3-in-1 rapid blood self-testing for HIV, hepatitis B and hepatitis C | Yes | Yes | Yes | Yes | Yes | No | Yes | Yes | Include |
| Sarkar et al. | 2016 | Feasibility of supervised self-testing using an oral fluid-based HIV rapid testing method: a cross-sectional, mixed method study among pregnant women in rural India | Yes | Yes | Yes | Yes | No | No | Yes | Yes | Include |
| Sayabounthavong et al. | 2019 | Optimizing individual and public health benefits of assisted HIV self-testing through effective linkages to services for men who have sex with men and transgender women in Laos | Unclear | Unclear | Yes | No | Unclear | Unclear | No | Unclear | Exclude |
| Shahmanesh et al. | 2019 | Mafrica: Zenzele, a mobile-phone enabled HIV testing and linkage to care pathway for young people in rural South Africa | No | No | Unclear | Unclear | Unclear | Unclear | Unclear | Yes | Include |
| Shally et al. | 2020 | Lessons learned from transgender women peers offering HIV-self testing and assisted partner notification services in Malindi, Kenya | Yes | Yes | Yes | Yes | Unclear | Unclear | Yes | Unclear | Exclude |
| Sithole et al. | 2022 | Secondary distribution of HIV self-testing kits to social and sexual networks of PLWH in KwaZulu-Natal, South Africa. a brief report | Yes | Yes | Unclear | Unclear | No | No | Unclear | Yes | Include |
| Sithole et al. | 2021 | Implementation of HIV self-testing to reach men in rural uMkhanyakude, KwaZulu-Natal, South Africa. a DO-ART trial sub study | Yes | Yes | Yes | Yes | Yes | Yes | Yes | Yes | Include |
| Stafylis et al. | 2018 | Vending machines in commercial sex venues to increase HIV self-testing among men who have sex with men | Yes | Yes | Yes | Yes | No | No | Yes | Yes | Include |
| Stafylis, et al. | 2022 | Relative Effectiveness of Social Media, Dating Apps, and Information Search Sites in Promoting HIV Self-testing: Observational Cohort Study | Yes | Yes | Yes | Yes | Yes | No | Yes | Yes | Include |
| Tama et al. | 2021 | Does onsite HIV self-testing enhance linkage to care? A case study of HIVST Challenge Fund Project in Kenya | Unclear | Unclear | Yes | Yes | Unclear | Unclear | Yes | Unclear | Exclude |
| Tang et al. | 2019 | What happens after HIV self-testing? Results from a longitudinal cohort of Chinese men who have sex with men | Yes | Yes | Yes | Yes | No | No | Yes | Yes | Include |
| Tao et al. | 2014 | Home-based HIV testing for men who have sex with men in China: A novel community-based partnership to complement government programs | Yes | Yes | Yes | Yes | Yes | Yes | Yes | Yes | Include |
| Thirumurthy et al. | 2016 | Promoting male partner HIV testing and safer sexual decision making through secondary distribution of self-tests by HIV-negative female sex workers and women receiving antenatal and post-partum care in Kenya: a cohort study | Yes | Yes | Yes | Yes | No | No | Yes | Yes | Include |
| Tonen-Wolyec et al. | 2019 | Acceptability, feasibility, and individual preferences of blood-based HIV self-testing in a population-based sample of adolescents in Kisangani, Democratic Republic of the Congo | Yes | Yes | Yes | Yes | No | No | Yes | Yes | Include |
| Trabwongwitaya, et al. | 2022 | Preference and ability to perform blood-versus oral-fluid-based HIV self-testing in adolescents and young adults in Bangkok | Yes | Yes | Yes | Yes | Yes | No | Yes | Yes | Include |
| Tukei et al. | 2019 | HIV self-test uptake, yield, and linkage experiences among key populations in LESOTHO | No | No | Yes | Yes | No | No | Yes | Yes | Include |
| Tumusiime et al. | 2022 | Peer-led community HIV self-testing (HIVST) distribution models improves access to HIV testing services (HTS) among key populations (KP) in Uganda | Yes | Yes | Yes | Yes | Unclear | Unclear | Yes | Unclear | Exclude |
| Tun et al. | 2018 | Uptake of HIV self-testing and linkage to treatment among men who have sex with men (MSM) in Nigeria: A pilot programme using key opinion leaders to reach MSM | Yes | Yes | Yes | Yes | No | No | Yes | Yes | Include |
| Udeagu et al. | 2017 | Men who have sex with men seek timely human immunodeficiency virus confirmation and care after rapid human immunodeficiency virus self-test: data from partner services program, new york city | Yes | Yes | Yes | Yes | No | No | Yes | Yes | Include |
| Vera et al. | 2019 | Acceptability and feasibility of using digital vending machines to deliver HIV self-tests to men who have sex with men | No | Yes | No | Yes | No | No | Yes | NA | Exclude |
| Villares et al. | 2021 | User assessment of HIV self-testing (HIVST) in Brazil: An acceptable tool with great potential for reaching key populations and maximize positivity yield | Yes | No | Yes | Yes | No | No | Yes | NA | Exclude |
| Volk et al. | 2016 | Acceptability and feasibility of HIV self-testing among men who have sex with men in Peru and Brazil | Yes | Yes | Yes | No | No | No | Yes | Yes | Include |
| Vu et al. | 2020 | Leading from the community: How key population organizations in Vietnam transformed from peer support groups to clinical service providers | Unclear | Unclear | Yes | Unclear | Unclear | Unclear | Yes | NA | Exclude |
| Wang et al. | 2020 | Correlates of HIV self-testing among female sex workers in China: implications for expanding HIV screening | Yes | Yes | Yes | Yes | No | No | Yes | Yes | Include |
| Wang et al. | 2020 | Promoting oral HIV self‐testing via the internet among men who have sex with men in China: a feasibility assessment | Yes | Yes | Yes | Yes | No | No | Yes | Yes | Include |
| Widyanthini et al. | 2022 | HIV self-testing for men who have sex with men: an implementation trial in Indonesia | Yes | Yes | No | Yes | No | No | Yes | Yes | Include |
| Wilson et al. | 2022 | High acceptance and completion of hiv self-testing among diverse populations of young people in kenya using a community-based distribution strategy | Yes | Yes | Yes | Yes | No | No | Yes | Yes | Include |
| Wu et al. | 2021 | Social Media–Based Secondary Distribution of Human Immunodeficiency Virus/Syphilis Self-testing Among Chinese Men Who Have Sex with Men | Yes | Yes | Yes | Yes | No | No | Yes | Yes | Include |
| Yang et al. | 2019 | HIV self-testing and potential linkage to care among men who have sex with men in China: A cross-sectional online survey | Yes | Yes | Yes | Yes | Unclear | Unclear | Yes | Yes | Include |
| Zhang et al. | 2020 | HIV self-testing programs to men who have sex with men delivered by social media key opinion leaders and community-based organizations are both effective and complementary: a national pragmatic study in China | Yes | Yes | No | Yes | No | No | Yes | Yes | Include |
| Zhang et al. | 2021 | The longer-term effects of access to HIV self-tests on HIV testing frequency in high-risk gay and bisexual men: follow-up data from a randomised controlled trial | Unclear | Yes | Unclear | Yes | No | No | Yes | Yes | Include |
| Zhong et al. | 2017 | Acceptability and feasibility of a social entrepreneurship testing model to promote HIV self-testing and linkage to care among men who have sex with men | Yes | Yes | Yes | No | No | No | Yes | Yes | Include |
| Zishiri, et al. | 2022 | Secondary distribution of HIV self-test kits by HIV index and antenatal care clients: implementation and costing results from the STAR Initiative in South Africa | Yes | Yes | Yes | No | No | No | Yes | Yes | Include |

AIDS, acquired immunodeficiency syndrome; ART, antiretroviral therapy; FSW, female sex workers; HIV, human immunodeficiency virus; HIVST, HIV self–testing; KP, key populations; MSM, men who have sex with men; NGO, nongovernmental organisation; PLWHIV, people living with HIV; PrEP, pre–exposure prophylaxis; PWID, people who inject drugs; RCT, randomised controlled trial; SNA, social network–based approach; STD, sexually transmitted diseases

## Figure S19. Summary graph for risk of bias of RCT studies


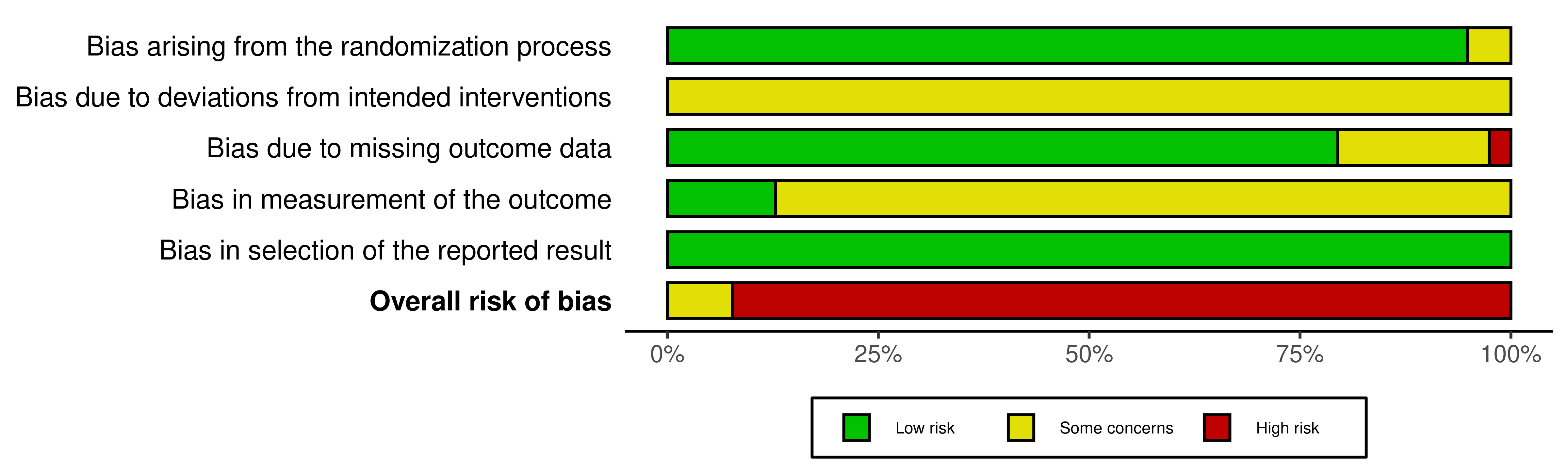


## Figure S20. Visual graph for risk of bias of RCT studies


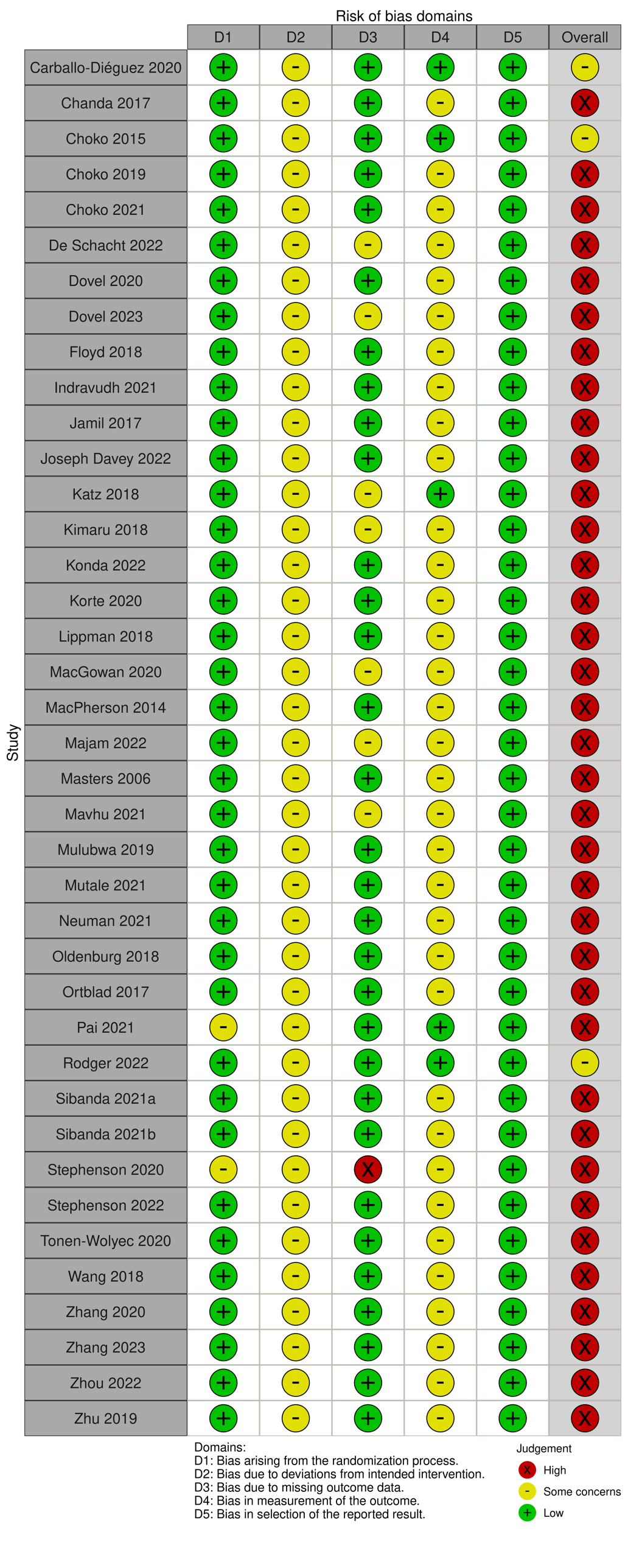

Supplement: Supplementary file 1 — Table S1. Literature search strategy Table S2. Summary of included studies Table S3. Quality assessment of qualitative studies Table S4. Quality assessment of randomised controlled trial studies Table S5. Quality assessment of quasi‐experimental studies Table S6. Quality assessment for cross‐sectional studies Figure S1. Forest plot for the proportion who were linked to confirmatory testing in unassisted HIVST Figure S2. Forest plot for proportion who were linked to confirmatory testing in assisted HIVST Figure S3. Forest plot for proportion who were linked to ART initiation reports in unassisted HIVST Figure S4. Forest plot for proportion who were linked to ART initiation reports in assisted HIVST Figure S5. Forest plot for proportion who were linked to confirmatory testing from health facility delivery model Figure S6. Forest plot for proportion who were linked to confirmatory testing from community delivery model Figure S7. Forest plot for proportion who were linked to confirmatory testing from pharmacy delivery model Figure S8. Forest plot for proportion who were linked to confirmatory testing from online/mail delivery model Figure S9. Forest plot for proportion who were linked to confirmatory testing from peer‐educator delivery model Figure S10. Forest plot for proportion who were linked to confirmatory testing from antenatal care delivery model Figure S11. Forest plot for proportion who were linked to confirmatory testing from other delivery models Figure S12. Forest plot for proportion who were linked to confirmatory testing with >1 type of delivery models Figure S13. Forest plot for proportion who were linked to ART initiation reports in RCT Figure S14. Forest plot for proportion who were linked to ART initiation reports in non‐RCT Figure S15. Doi plot of included studies for linkage to confirmatory testing Figure S16. Doi plot of included studies for linkage to ART initiation Figure S17. Doi plot of included studies for linkage to care Figure S18. Doi plot o [file JIA2-27-e26388-s001.docx]
